# Efficient oxidation of oleanolic acid derivatives using magnesium bis(monoperoxyphthalate) hexahydrate (MMPP): A convenient 2-step procedure towards 12-oxo-28-carboxylic acid derivatives

**DOI:** 10.3762/bjoc.8.17

**Published:** 2012-01-30

**Authors:** Jorge A R Salvador, Vânia M Moreira, Rui M A Pinto, Ana S Leal, José A Paixão

**Affiliations:** 1 Grupo de Química Farmacêutica, Faculdade de Farmácia da Universidade de Coimbra, Pólo das Ciências da Saúde, Azinhaga de Santa Comba, 3000-548, Coimbra, Portugal; 2 Centro de Neurociências e Biologia Celular, Universidade de Coimbra, 3004-517 Coimbra, Portugal; 3 Centro de Química de Coimbra, Faculdade de Ciências e Tecnologia da Universidade de Coimbra, Rua Larga, 3004-535 Coimbra, Portugal; 4 CEMDRX, Departamento de Física, Faculdade de Ciências e Tecnologia, Universidade de Coimbra, 3004-516 Coimbra, Portugal

**Keywords:** bismuth(III) triflate, δ-hydroxy-γ-lactones, MMPP, oleanolic acid, triterpenoid

## Abstract

A new, straightforward and high yielding procedure to convert oleanolic acid derivatives into the corresponding δ-hydroxy-γ-lactones, by using the convenient oxidizing agent magnesium bis(monoperoxyphthalate) hexahydrate (MMPP) in refluxing acetonitrile, is reported. In addition, a two-step procedure for the preparation of oleanolic 12-oxo-28-carboxylic acid derivatives directly from Δ^12^-oleananes, without the need for an intermediary work-up, and keeping the same reaction solvent in both steps, is described as applied to the synthesis of 3,12-dioxoolean-28-oic acid.

## Findings

The molecular diversity that arises from research into natural products represents a valuable tool for driving drug discovery and development [1–2]. In this context, pentacyclic triterpenoids are currently regarded as important scaffolds for new drug development [3]. The chemistry of oleanane-type triterpenoids has been investigated with particular interest and many relevant biological and pharmacological activities of these derivatives have been reported in the literature, among which are antitumor, antiviral, anti-inflammatory, hepatoprotective, gastroprotective, antimicrobial, antidiabetic, and hemolytic properties, as well as many others [3–5]. Functionalized γ-lactones are important building blocks of bioactive natural products [6–7]. The δ-hydroxy-γ-lactone motif is part of such bioactive natural products as (±)-muricatacin [8–9] or the three hydroxylactones found in the mushroom *Mycoleptodonoides aitchisonii* [10]. Terpenoid δ-hydroxy-γ-spirolactones have been found to act as significant feeding deterrents to the lesser mealworm *Alphitobius diaperinus* [11]. In particular, oleanane-type triterpenoids bearing a γ-lactone function, either isolated from natural sources or obtained by semisynthesis, have shown interesting biological activities [12–14]. From the synthetic point of view, the oxidative 28,13β-lactonization allows the preparation of 12α-hydroxyoleananes with a protected 28-carboxyl acid function. In fact, 12α-hydroxy-3-oxooleanan-28,13β-olide (**2**) is a key intermediate in the synthesis of S-0139, an endothelin A receptor antagonist [15]. Moreover, as part of our ongoing work on pentacyclic triterpenoid chemistry [16–17], we recently demonstrated that oleanolic δ-hydroxy-γ-lactones can be efficiently converted into the corresponding 12-oxo-28-carboxylic acid derivatives by bismuth(III) triflate catalysis [18]. This new approach not only avoids an inconvenient multistep synthesis by means of a protection/deprotection strategy [19–20] but also results in chemical modification of ring C, a strategy known to increase the anti-inflammatory and cytotoxic activities of oleanolic acid (OA) derivatives [19,21–22].

Oleanolic δ-hydroxy-γ-lactones can be obtained from Δ^12^-oleananes by oxidative 28,13β-lactonization. This reaction was performed under photochemical irradiation [23–24], but weak selectivity and low isolated yields were observed. Alternatively, oxidation reagents such as H_2_O_2_ in acetic acid [25–26], the inorganic salt mixture KMnO_4_/CuSO_4_ [27], ozone [15,28–29] and *m-*chloroperoxybenzoic acid (mCPBA) [30–31] have also been reported. Magnesium bis(monoperoxyphthalate) hexahydrate (MMPP) is commercially available, inexpensive and relatively stable [32–34] and has been used in the oxidation of various functional groups [35–42]. This oxidant is non-shock-sensitive and non-deflagrating [43]. Moreover, its use greatly simplifies the isolation of the reaction products, because it may simply be filtered off from the reaction crude, which is then worked up as usual.

In this letter, we report the use of MMPP for the efficient and high-yielding oxidation of OA derivatives to afford the corresponding δ-hydroxy-γ-lactones. Moreover, we have set up a protocol that allows the convenient sequential two-step preparation of 3,12-dioxoolean-28-oic acid directly from 3-oxooleanolic acid, without the need of an intermediary work-up, and keeping the same reaction solvent in both steps.

We found that the reaction of 3-oxooleanolic acid **1** with 2.0 equiv of MMPP, in refluxing acetonitrile, afforded the corresponding δ-hydroxy-γ-lactone **2** in 85% yield after 5 hours (Table 1, entry 1). These new reaction conditions were successfully extended to OA **3** and other 3β-substituted OA derivatives **5**, **7** and **9** (Table 1, entries 2–5).

**Table 1:** Reaction of OA derivatives with MMPP to afford oleanolic δ-hydroxy-γ-lactones directly.^a^

Entry	Substrate (mmol)	MMPP (equiv)	Time (h)	Product^b^	Yield^c^ (%)

1	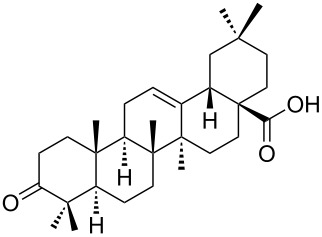 **1** (0.16 mmol)	2.0	5	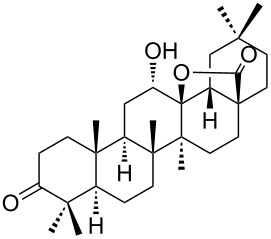 **2**	85
2	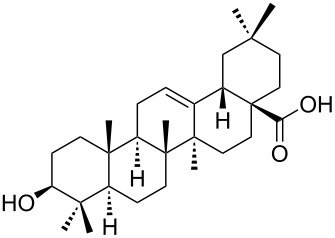 **3** (0.16 mmol)	2.0	24	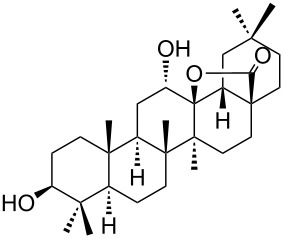 **4**	84
3	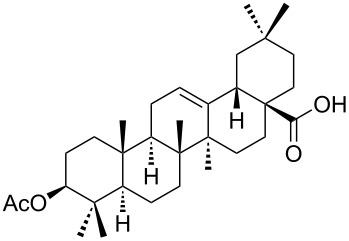 **5** (0.15 mmol)	2.0	8	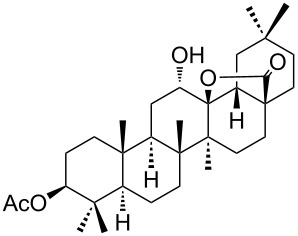 **6**	88
4	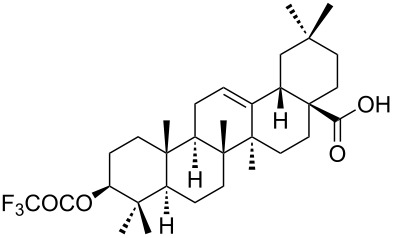 **7** (0.13 mmol)	2.6	24	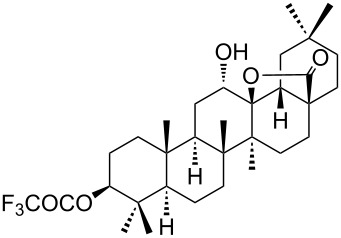 **8**	98
5	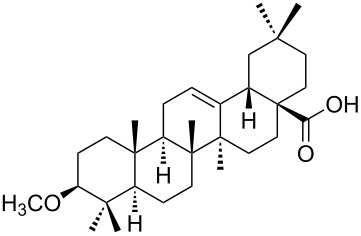 **9** (0.16 mmol)	3.0	24	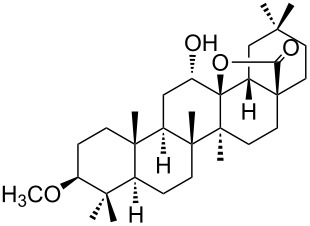 **10**	92

^a^Reactions were performed in acetonitrile, under reflux; ^b^Analytical data for compounds **2** [29], **4** [28], **6** [27], **8** [18] and **10** [18] are in accordance with the literature; ^c^Isolated yield.

The substrates were dissolved in acetonitrile under reflux, and MMPP (2.0–3.0 equiv) was added to the solution under strong magnetic stirring. After completion of the reaction, the magnesium salts were easily filtered off after evaporation of the acetonitrile and suspension of the resulting white solid in ethyl acetate. We found that 2.0 equiv of MMPP were sufficient to effectively convert 3-oxooleanolic acid **1**, OA **3**, and 3β-acetoxyoleanolic acid **5** into the corresponding δ-hydroxy-γ-lactones **2**, **4** and **6**, in 84 to 88% yield (Table 1, entries 1–3). Substrates **7** and **9**, bearing a trifluoroacetoxy and a methoxy group at C3, respectively, required higher amounts of the reagent and longer reaction times (Table 1, entries 4 and 5). The formation of the oleanolic δ-hydroxy-γ-lactones **2**, **4**, **6**, **8** and **10**, may be explained by epoxidation of the parent Δ^12^-oleanane compound, followed by nucleophilic attack of the 28-carboxyl group at C13 from the β-face, with ring-opening of the 12α,13α-epoxide intermediate [15,44].

**Figure 1:**
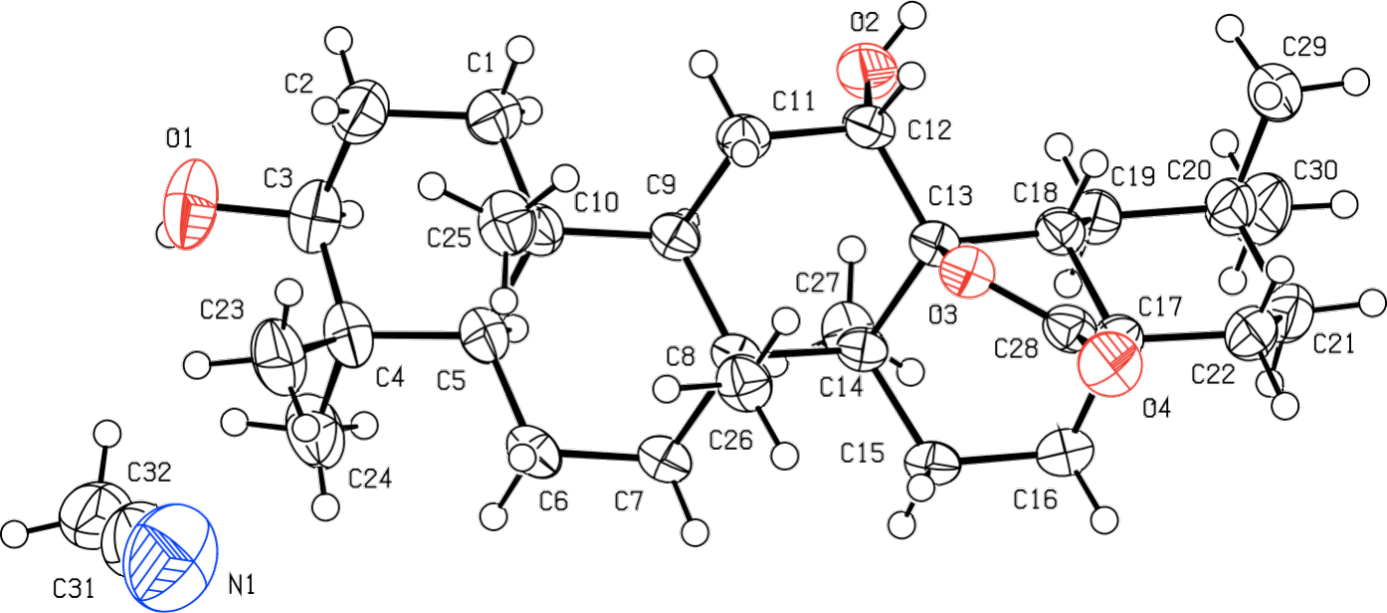
ORTEP diagram of compound **4** (50% probability level, H atoms of arbitrary sizes). The asymmetric unit also contains a molecule of CH_3_CN.

Quite recently, we demonstrated that the 28,13β-lactonization of 3-oxooleanolic acid **1** promoted by Bi(OTf)_3_·xH_2_O affords a 3-oxo-18α-olean-28,13β-olide product, with inversion of configuration at the C18-stereocenter, as demonstrated by X-ray crystallography [45–46]. In order to assign the orientation of the 18-H of the 12α-hydroxy-γ-lactones obtained in this work, 2D NMR data were collected for compounds **2**, **6** and **8**, and X-ray data were gathered for compound **4**. Combining 1D and 2D-NMR spectroscopy, we were able to determine the chemical shift of 18-H (2.02 ppm) for compound **2**. This value is much lower than the one of the parent substrate **1** (2.84 ppm), which may be explained by magnetic anisotropy induced by the 28,13β-lactone moiety. It is also interesting to note that a long-distance coupling between the 18-H and 12β-H (3.90 ppm) signals was found in the COSY spectrum of **2**. Correlation between these two signals was also observed in the NOESY experiment and, therefore, the β-configuration was assigned at the C18-stereocenter. The same NMR pattern was present for compounds **6** and **8**. Unequivocal evidence of the molecular structure of compound **4** was obtained by single-crystal X-ray crystallography, and the ORTEP diagram with the corresponding atomic numbering scheme is depicted in Figure 1.

**Scheme 1:**
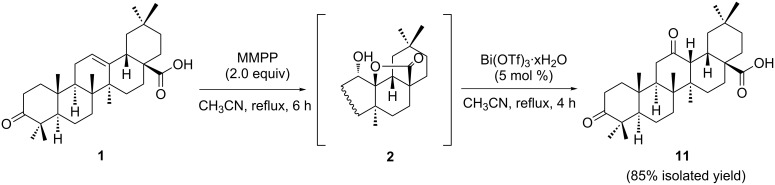
Sequential 2-step synthesis of 3,12-dioxoolean-28-oic acid (**11**) directly from 3-oxooleanolic acid (**1**).

In the past few years, bismuth(III) salts have emerged as convenient reagents for the development of new chemical processes under more “ecofriendly” reaction conditions, which avoid the use of large amounts of toxic and corrosive materials [47–51]. Bearing in mind the solubility properties of MMPP and that both the oxidative 28,13β-lactonization and the bismuth(III) triflate-catalyzed direct opening of δ-hydroxy-γ-lactones are performed in acetonitrile, we designed a protocol to perform the synthesis of oleanolic 12-oxo-28-carboxylic acid derivatives directly from Δ^12^-oleananes, without the need for an intermediary work-up, and keeping the same reaction solvent in both steps (Scheme 1).

**Figure 2:**
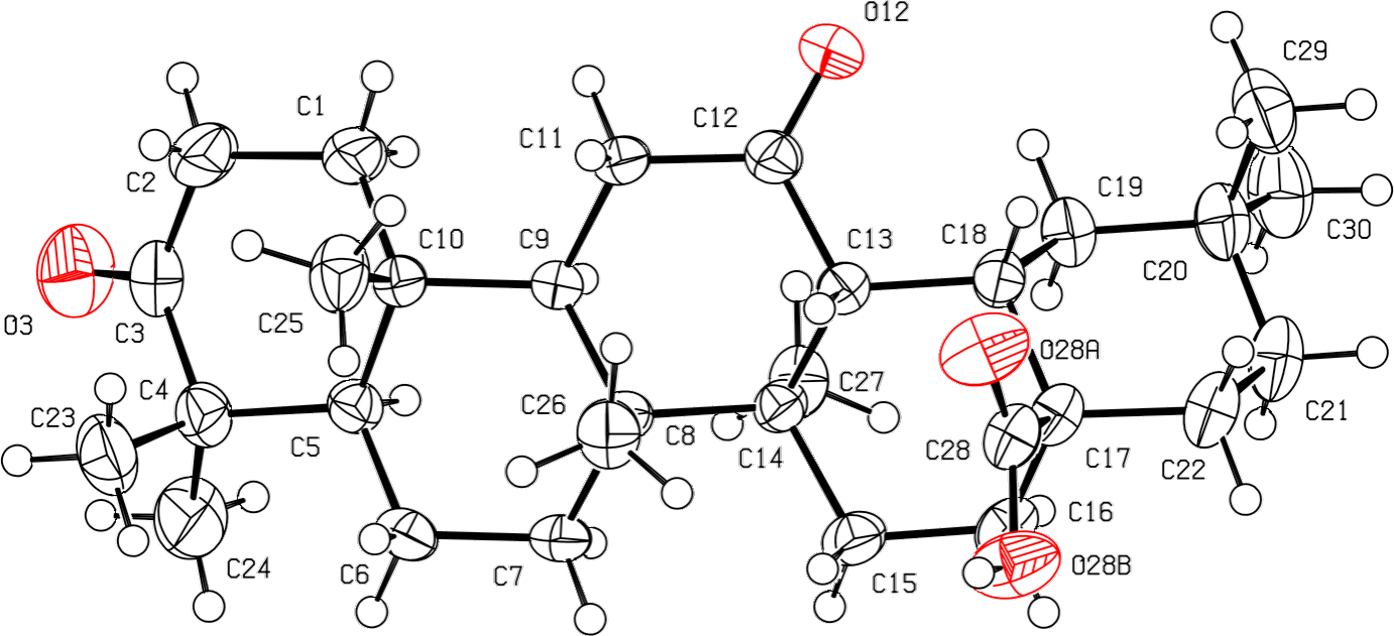
ORTEP diagram of compound **11** (50% probability level, H atoms of arbitrary sizes).

Thus, after the formation of the 12α-hydroxy-28,13β-olide compound **2** by MMPP oxidation, a filtration step allowed the elimination of insoluble magnesium salts, taking advantage of their low solubility in acetonitrile. Then, a catalytic amount of bismuth(III) triflate (5 mol %) was added to the resulting filtrate, and the expected 12-oxo-28-carboxylic acid **11** was obtained, in 85% yield, after the typical work-up procedure [18]. The formation of compound **11** from the 12α-hydroxy-28,13β-olide **2** is likely to occur due to the in situ generation of a Brønsted acid species from bismuth(III) triflate, which promotes ring opening of the 28,13β-olide group, creating a tertiary carbocation at C-13. Then, a concerted stereoselective 1,2-migration of the 12β-H to the 13β-position with the rearrangement of the 12α-hydroxy group affords the final 12-oxo-28-carboxylic acid structure [18]. The molecular structure of compound **11**, determined by single-crystal X-ray crystallography, is shown in Figure 2.

In conclusion, we have found a new straightforward procedure to convert OA derivatives into δ-hydroxy-γ-lactones, in very high yields, using the convenient oxidizing agent MMPP. This procedure has considerable advantages over the previously reported oxidation methods, because no other positions of the molecule are oxidized concomitantly, it avoids the use of halogenated solvents, and allows easy recovery of the reaction products. Combination of this oxidative 28,13β-lactonization process with the ability of bismuth(III) triflate to catalyze the opening of the resulting δ-hydroxy-γ-lactone with subsequent generation of the carbonyl group, allowed us to set up a sequential two step strategy for the preparation of 3,12-dioxoolean-28-oic acid (**11**) directly from 3-oxooleanolic acid **1**, that avoids an intermediary work-up and conveniently uses the same reaction solvent in both steps. Thus, the procedure reported herein greatly simplifies the obtainment of oleanolic δ-hydroxy-γ-lactones, which are versatile intermediates for organic synthesis, and in addition can provide very easy access to the corresponding oleanolic 12-oxo-28-carboxylic acids.

## Supporting Information

The Supporting Information contains the typical procedure for the MMPP oxidative 28,13β-lactonization and preparation of compounds **2**, **4**, **6**, **8** and **10**. Moreover, the procedure for the sequential two step synthesis of 3,12-dioxoolean-28-oic acid (**11**) is described and the 1D and 2D NMR spectra of compounds **2**, **4**, **6**, **8**, **10** and 1D NMR spectra of compound **11** are shown.

File 1 Experimental and analytical data.
