# Supplementary material for: Efficient oxidation of oleanolic acid derivatives using magnesium bis(monoperoxyphthalate) hexahydrate (MMPP): A convenient 2-step procedure towards 12-oxo-28-carboxylic acid derivatives
Source: Beilstein J Org Chem. 2012 Jan 30;8:164–9. doi: 10.3762/bjoc.8.17 (PMC3302076; doi:10.3762/bjoc.8.17)
Supplement: File 1 — Experimental and analytical data. [file Beilstein_J_Org_Chem-08-164-s001.pdf]

Supporting Information
for
Efficient oxidation of oleanolic acid derivatives using magnesium bis(monoperoxyphthalate) hexahydrate (MMPP): A convenient 2-step procedure towards 12-oxo-28-carboxylic acid derivatives

Jorge A. R. Salvador,*[,1,2] Vânia M. Moreira,[1,3] Rui M. A. Pinto,[1] Ana S. Leal[1] and José A. Paixão[4]

Address: [1]Grupo de Química Farmacêutica, Faculdade de Farmácia da Universidade de Coimbra, Pólo das Ciências da Saúde, Azinhaga de Santa Comba, 3000-548, Coimbra, Portugal; [2]Centro de Neurociências e Biologia Celular, Universidade de Coimbra, 3004-517 Coimbra, Portugal; [3]Centro de Química de Coimbra, Faculdade de Ciências e Tecnologia da Universidade de Coimbra, Rua Larga, 3004-535 Coimbra, Portugal and [4]CEMDRX, Departamento de Física, Faculdade de Ciências e Tecnologia, Universidade de Coimbra, 3004-516 Coimbra, Portugal

Email: Jorge A. R. Salvador* - salvador@ci.uc.pt

* Corresponding author

Experimental and analytical data

Table of contents

Typical procedure for the MMPP oxidative 28,13β-lactonization..S3
3β,12α-Dihydroxyolean-28,13β-olide (**4**)..S4
3β-Acetoxy-12α-hydroxyolean-28,13β-olide (**6**)...S4
3β-Trifluoroacetoxy-12α-hydroxyolean-28,13β-olide (**8**)..S4
12α-Hydroxy-3β-methoxyolean-28,13β-olide (**10**)..S5
Procedure for the sequential two-step synthesis of 3,12-dioxoolean-28-oic acid (**11**).............S5
1D and 2D NMR data for compound **2**...S7
1D NMR data for compound **4**..S14
1D and 2D NMR data for compound **6**..S17
1D and 2D NMR data for compound **8**..S24
1D NMR data for compound **10**..S29
1D NMR data for compound **11**..S32
References...S35

All reagents, including OA **3**, were obtained from Sigma–Aldrich Co. OA derivatives **1** [1], **5** [2], **7** [3] and **9** [4] were synthesized according to literature procedures. For thin layer chromatography (TLC) analysis, Kieselgel 60HF254/Kieselgel 60G was used. IR spectra were obtained on a JASCO FT/IR-420 spectrophotometer (FTIR-ATR). NMR spectra were obtained on a Bruker Digital NMR Avance 400 spectrometer, in $CDCl_3$ with Me_4Si as the internal standard. Mass spectra were recorded on a Finnigan PolarisQGC/MS Benchtop Ion Trap mass spectrometer. Single-crystal X-ray diffractometry analysis was made on a Bruker-Nonius Kappa Apex II CCD diffractometer employing graphite monochromated Mo Kα radiation (λ = 0.71073 Å). The structure was solved by direct methods and conventional Fourier synthesis (SHELXS-97). The refinement of the structure was made by full matrix least-squares on *F*2 (SHELXL-97). All non-H-atoms were refined anisotropically. The H atoms positions were initially placed at idealized calculated positions and refined with isotropic thermal factors while being allowed to ride on the attached parent atoms by using SHELXL-97 defaults. Crystallographic data have been deposited at the Cambridge Crystallographic Data Center (CCDC deposition numbers 809124 and 844828, respectively, for compounds **4** and **11**). Copies can be obtained, free of charge, from CCDC, 12 Union Road, Cambridge CB2 1EZ, UK; fax: +44 1223 336033; e-mail: deposit@ccdc.cam.ac.uk).

Typical procedure for the MMPP oxidative 28,13β-lactonization: To a solution of 3-oxo-oleanolic acid (**1**, 75 mg, 0.16 mmol) in acetonitrile (5.7 mL) at reflux, MMPP (197 mg, 0.32 mmol) was added, and the reaction mixture was stirred for 5 h. The reaction solvent was then removed under reduced pressure, and the resulting mixture was suspended in ethyl acetate (75 mL) and filtered. The filtrate was washed with 10% aqueous $NaHCO_3$ (20 mL), 10% aqueous Na_2SO_3 solution (2 × 20 mL), water (20 mL) and brine (20 mL). The organic phase was dried with anhydrous Na_2SO_4, filtered, and concentrated under reduced pressure to give 12α-hydroxy-3-oxoolean-28,13β-olide **2** as a white solid (65.6 mg, 85%). Mp (MeOH): 283–285 °C (dec.) [1]. IR (film) 3494, 1750, 1703 cm^{-1}; ^1H NMR (400 MHz, $CDCl_3$) δ = 0.89 (s, 3H), 0.97 (s, 6H), 1.03 (s, 3H), 1.09 (s, 3H), 1.18 (s, 3H), 1.31 (s, 3H), 3.90 (m, 1H, 12β-H); ^{13}C NMR (100 MHz, $CDCl_3$) δ = 16.3, 18.2, 18.4, 19.1, 21.1, 21.2, 23.9, 26.7, 27.5, 28, 29.1, 31.6, 33.3, 33.4, 34, 34.1, 36.2, 39.5, 39.6, 42.2, 42.2, 43.8, 44.7, 47.4, 51.2, 54.8, 76.1 (C12), 90.7 (C13), 180 (C28), 217.8

(C3); MS (EI): *m/z* (%) = 471 (5) $[M + H]^+$, 249 (9), 234 (27), 205 (100), 189 (56), 147 (45), 119 (51), 90 (48).

3β,12α-Dihydroxyolean-28,13β-olide (**4**): Obtained from OA **3** in 84% yield, after 24 h under the reaction conditions described above. Recrystallization from CH_3CN at rt afforded colourless single crystals suitable for X-ray crystallography. Compound **4** crystallizes in triclinic cell, *P*-1 space group, and its structure was refined down to a *R*1 = 0.0368 for 2772 reflections with $I > 2\sigma(I)$, 344 parameters and $wR(F^2)$ was 0.0993 (all data, 3133 reflections). Mp > 300 °C (dec.) [5]. IR (film) 3442, 1751 cm^{-1}; ^1H NMR (400 MHz, $CDCl_3$) δ = 0.77 (s, 3H), 0.87 (s, 3H), 0.89 (s, 3H), 0.97 (s, 3H), 0.98 (s, 3H), 1.14 (s, 3H), 1.30 (s, 3H), 3.21 (dd, J_1 = 5 Hz and J_2 = 11.2 Hz, 1H, 3α-H), 3.88 (m, 1H, 12β-H); ^{13}C NMR (100 MHz, $CDCl_3$) δ = 15.3, 16.3, 17.7, 18.5, 18.6, 21.2, 23.9, 27.2, 27.4, 28, 28, 28.7, 31.5, 33.2, 33.9, 34.1, 36.4, 38.8, 38.9, 39.4, 42, 42.3, 44.5, 44.7, 51.1, 55.2, 76.3 (C12), 78.8 (C3), 90.6 (C13), 180 (C28); MS (EI): *m/z* (%) = 473 (17) $[M + H]^+$, 217 (50), 206 (68), 189 (100), 147 (53), 119 (65), 105 (60), 78 (51).

3β-Acetoxy-12α-hydroxyolean-28,13β-olide (**6**): Obtained from 3β-acetoxyoleanolic acid **5** in 88% yield, after 8 h under the above described reaction conditions. Mp (ethyl acetate:petroleum ether) 285–287 °C [6]. IR (film) 3529, 1734, 1247 cm^{-1}; ^1H NMR (400 MHz, $CDCl_3$) δ = 0.84 (s, 3H), 0.86 (s, 3H), 0.89 (s, 6H), 0.97 (s, 3H), 1.13 (s, 3H), 1.30 (s, 3H), 2.04 (s, 3H, 3β-$OCOCH_3$), 3.87 (m, 1H, 12β-H), 4.47 (m, 1H, 3α-H); ^{13}C NMR (100 MHz, $CDCl_3$) δ = 16.2, 16.4, 17.6, 18.5, 18.5, 21.2, 21.3, 23.5, 23.8, 27.4, 27.9, 28, 28.7, 31.5, 33.2, 33.9, 34.1, 36.3, 37.8, 38.4, 39.2, 42, 42.3, 44.4, 44.7, 51, 55.2, 76.1 (C12), 80.9 (C3), 90.8 (C13), 171.2 ($O\underline{C}OCH_3$), 180.1 (C28); MS (EI): *m/z* (%) = 514 (6) M^+, 299 (18), 203 (53), 188 (100), 147 (34), 119 (43), 105 (34), 90 (27).

3β-Trifluoroacetoxy-12α-hydroxyolean-28,13β-olide (**8**): Obtained from 3β-trifluoroacetoxy oleanolic acid **7** in 98% yield, after 24 h under the reaction conditions described above. Mp (acetonitrile) 298–300 °C [3]. IR (film) 3529, 1770, 1735, 1167 cm^{-1}; ^1H NMR (400 MHz, $CDCl_3$) δ = 0.90 (s, 3H), 0.91 (s, 6H), 0.93 (s, 3H), 0.98 (s, 3H), 1.15 (s, 3H), 1.31 (s, 3H), 3.90 (m, 1H, 12β-H), 4.67 (dd, J_1 = 5.5 Hz and J_2 = 10.7 Hz, 1H, 3α-H); ^{13}C NMR (100 MHz, $CDCl_3$) δ = 16.2,

16.3, 17.5, 18.5, 18.5, 21.2, 23.1, 23.9, 27.4, 27.8, 28, 28.8, 31.6, 33.2, 33.8, 34.1, 36.3, 38.1, 38.3, 39.4, 42.1, 42.3, 44.4, 44.7, 51.1, 55.1, 76.1 (C12), 86 (C3), 90.4 (C13), 113.2 and 116.1 (C̲F_3, J = 286 Hz), 157.2 and 157.6 (OC̲OCF_3, J = 42 Hz), 179.9 (C28); MS (EI): *m/z* (%) = 568 (10) M^+, 507 (39), 263 (45), 218 (100), 188 (89), 177 (60), 118 (63), 104 (57).

12α-Hydroxy-3β-methoxyolean-28,13β-olide (**10**): Obtained from 3β-methoxyoleanolic acid **9** in 92 % yield, after 24 h under the reaction conditions described above. Mp (THF/MeOH) 279–281°C [3]. IR (film) 3524, 1739 cm^{-1}; ^1H NMR (400 MHz, $CDCl_3$) δ = 0.75 (s, 3H), 0.87 (s, 3H), 0.90 (s, 3H), 0.97 (s, 3H), 0.98 (s, 3H), 1.14 (s, 3H), 1.30 (s, 3H), 2.66 (dd, J_1 = 4.2 Hz and J_2 = 11.7 Hz, 1H, 3α-H), 3.35 (s, 3H, OCH_3), 3.88 (m, 1H, 12β-H); ^{13}C NMR (100 MHz, $CDCl_3$) δ = 16.1, 16.2, 17.6, 18.5, 18.6, 21.2, 22.1, 23.9, 27.4, 28, 28.8, 31.5, 33.2, 33.9, 34.1, 36.4, 38.7, 38.8, 39.4, 42, 42.3, 44.6, 44.7, 51.2, 55.7, 57.5, 76.4 (C12), 88.5 (C3), 90.6 (C13), 179.9 (C28); MS (EI): *m/z* (%) = 486 (20) M^+, 299 (23), 220 (33), 204 (45), 188 (100), 121 (39), 107 (51), 79 (46).

Procedure for the sequential two-step synthesis of 3,12-dioxoolean-28-oic acid (**11**): To a solution of 3-oxo-oleanolic acid **1** (150 mg, 0.32 mmol) in acetonitrile (12 mL), under reflux, MMPP (394 mg, 0.64 mmol) was added, and the reaction mixture was stirred for 6 h. It was then cooled to room temperature, filtered, and the filtrate washed with acetonitrile (2 mL). To the resulting clear solution, $Bi(OTf)_3 \cdot xH_2O$ (11.6 mg, 0.016 mmol) was added. After 4 h of stirring under reflux, the reaction was completed, as verified by TLC control. The reaction mixture was concentrated under reduced pressure. Ethyl acetate (40 mL) and water (20 mL) were added and the aqueous phase was further extracted with ethyl acetate (2 × 60 mL). The organic phase was washed with 10% aqueous $NaHCO_3$ solution (2 × 40 mL), water (40 mL) and brine (40 mL), dried with anhydrous Na_2SO_4, filtered, and concentrated under reduced pressure to give 3,12-dioxo-olean-28-oic acid **11** as a white solid (128 mg, 85%). Recrystallization from CH_3OH at rt afforded colourless single crystals suitable for X-ray crystallography. Compound **11** crystallizes in orthorhombic cell, $P2_12_12_1$ space group, and its structure was refined down to a $R1$ = 0.0483 for 3211 reflections with $I > 2\sigma(I)$, 315 parameters and $wR(F^2)$ was 0.1246 (all data, 3607 reflections). Mp 276–278 °C [3]. IR (film) 1700, 1698, 1696 cm^{-1}; ^1H NMR (400 MHz, $CDCl_3$) δ =

0.91 (s, 3H), 0.96 (s, 3H), 0.98 (s, 6H), 1.03 (s, 3H), 1.04 (s, 3H), 1.09 (s, 3H), 2.71 (d, 1H, J = 4.1 Hz, 13β-H), 2.77 (m, 1H, 18β-H); ^{13}C NMR (100 MHz, $CDCl_3$) δ. = 14.9, 16.1, 19.5, 20.4, 21.1, 22.6, 23.1, 26.3, 27.6, 30.6, 31.1, 31.8, 33, 33.3, 33.8, 34.4, 36.1, 36.6, 38.6, 41.2, 42, 47.1, 47.4, 49.1, 51.8, 54.8, 183.9 (C28), 210.9 (C12), 216.8 (C3); MS (EI): *m/z* (%) = 470 (8) M^+, 409 (56), 263 (41), 217 (100), 205 (64), 177 (55), 120 (35), 106 (49).

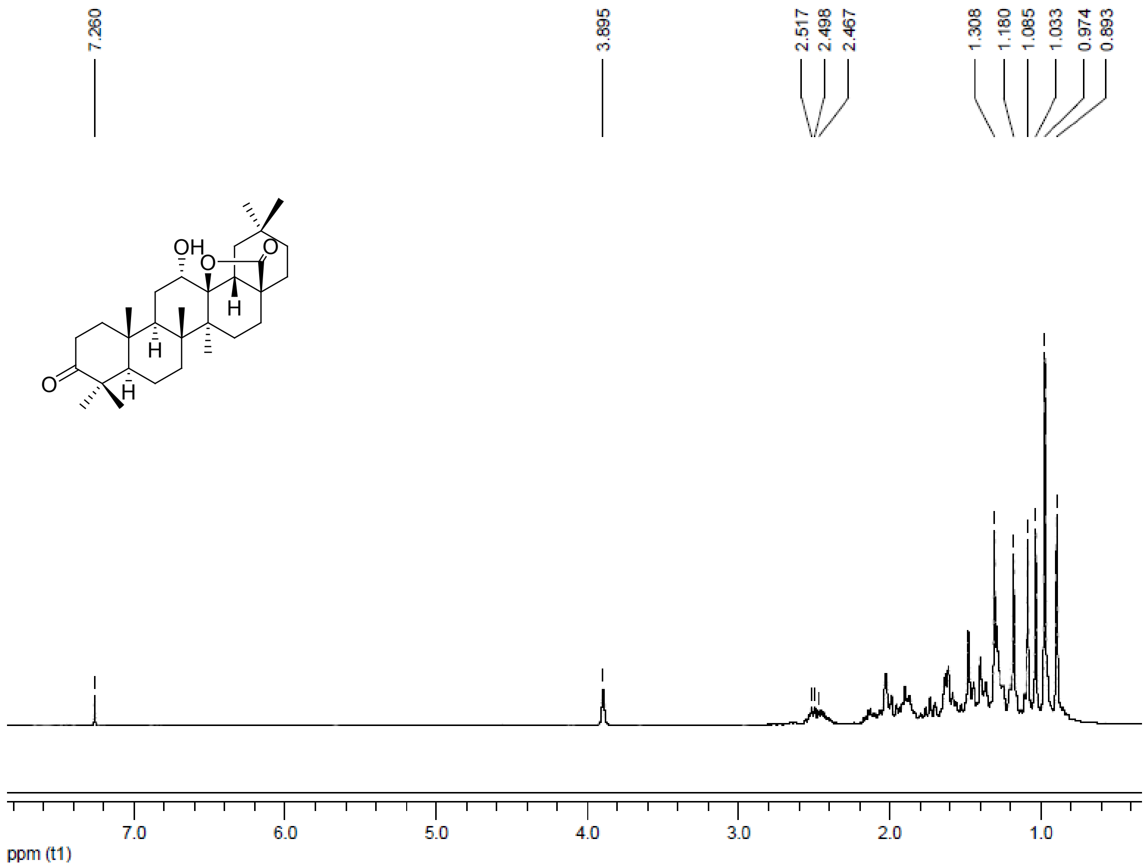

^{1}H NMR spectrum of compound **2** recorded in $CDCl_3$.

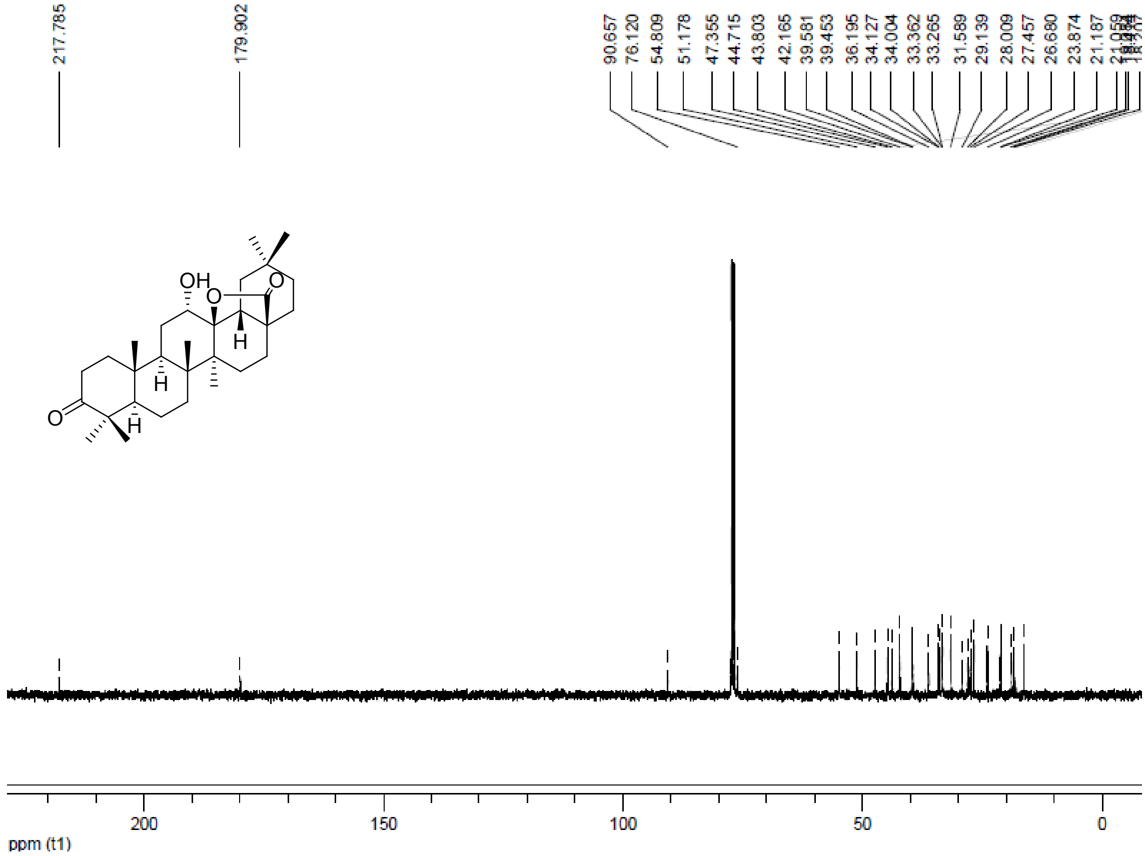

^{13}C NMR spectrum of compound **2** recorded in $CDCl_3$.

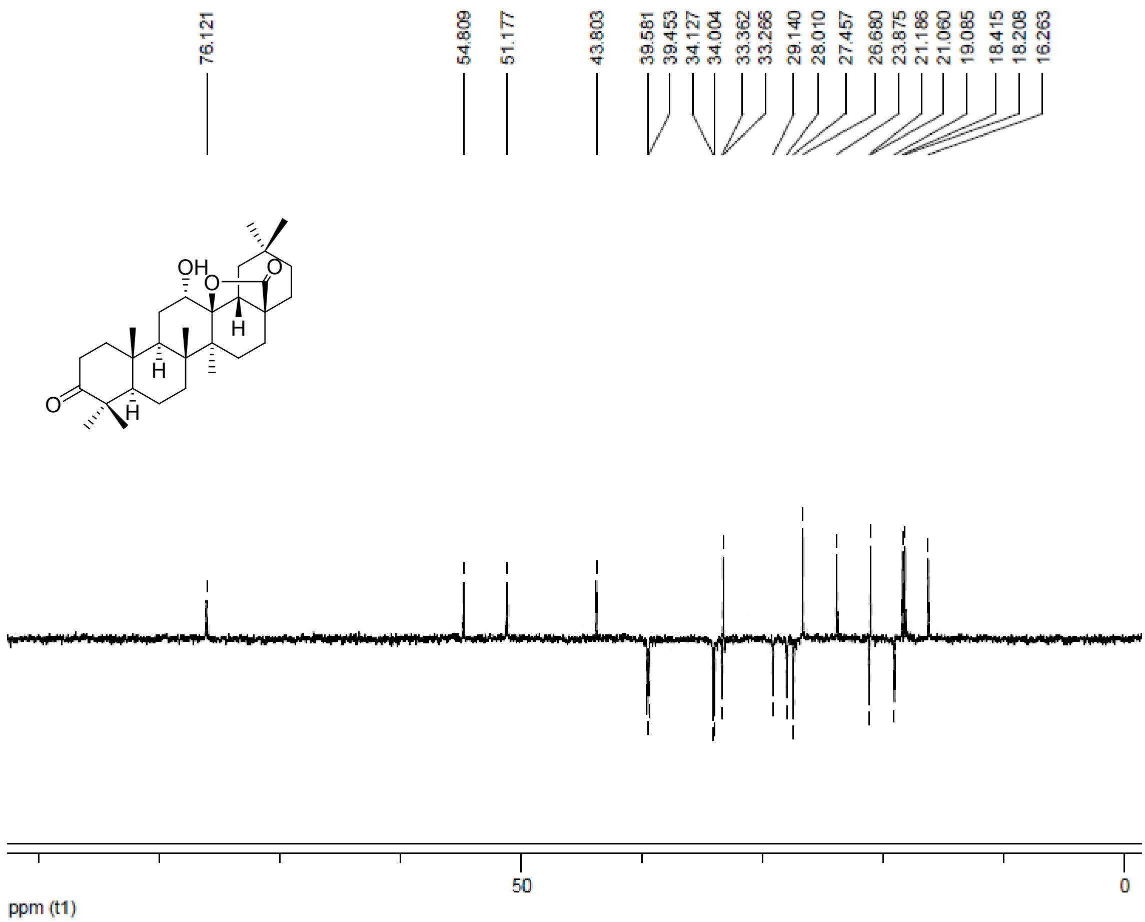

DEPT-135 spectrum of compound **2** recorded in $CDCl_3$.

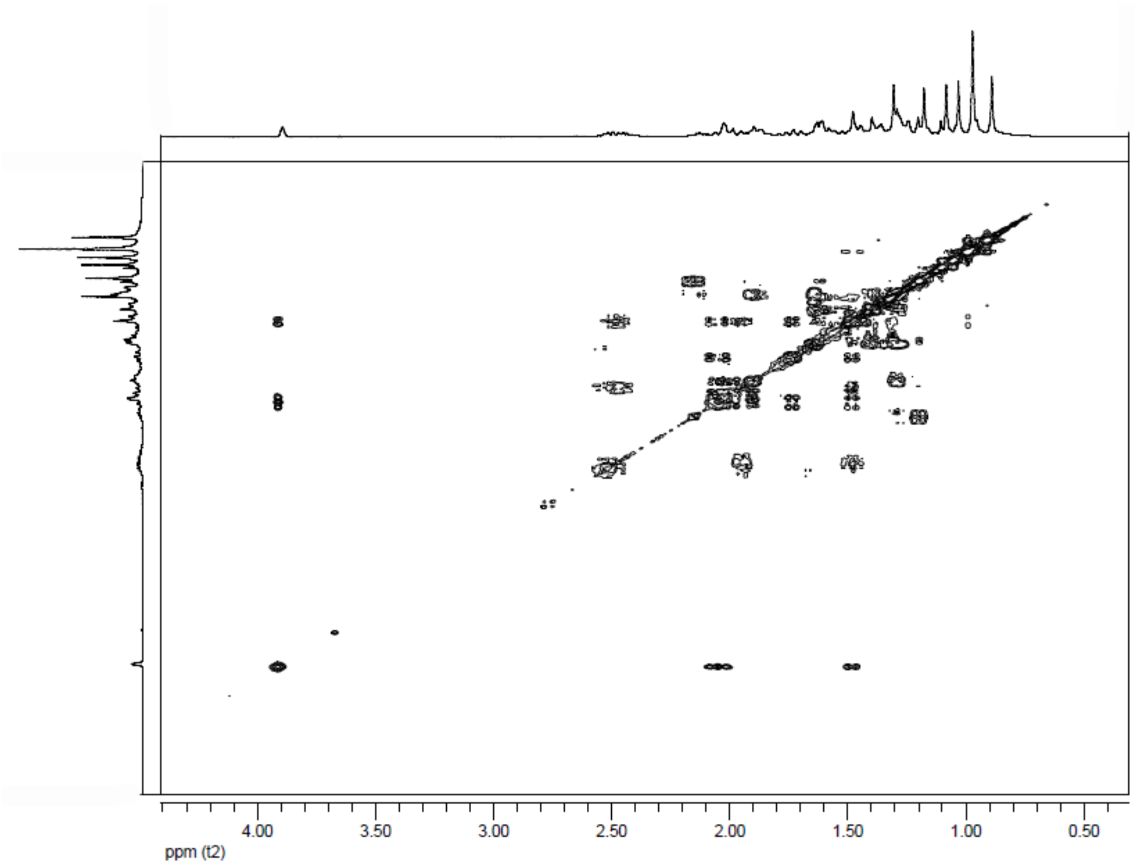

COSY spectrum of compound **2** recorded in $CDCl_3$.

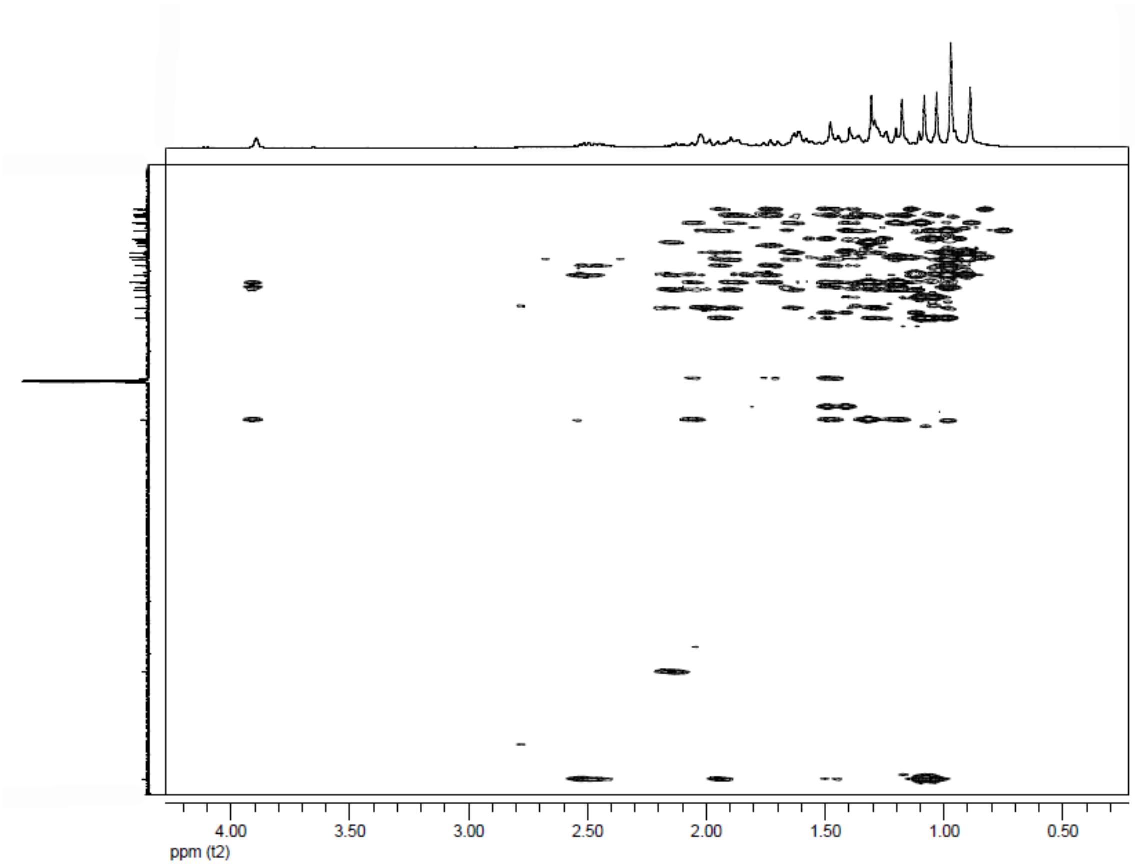

HMBC spectrum of compound **2** recorded in $CDCl_3$.

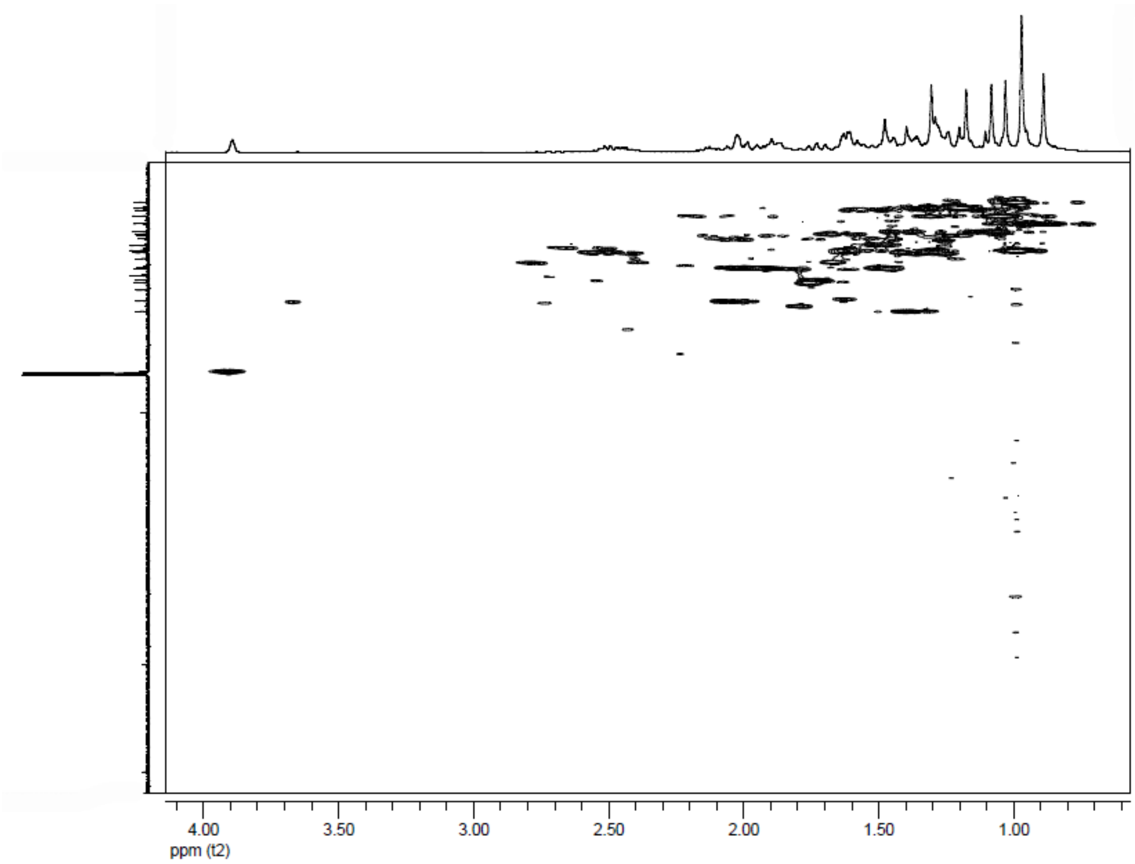

HMQC spectrum of compound **2** recorded in $CDCl_3$.

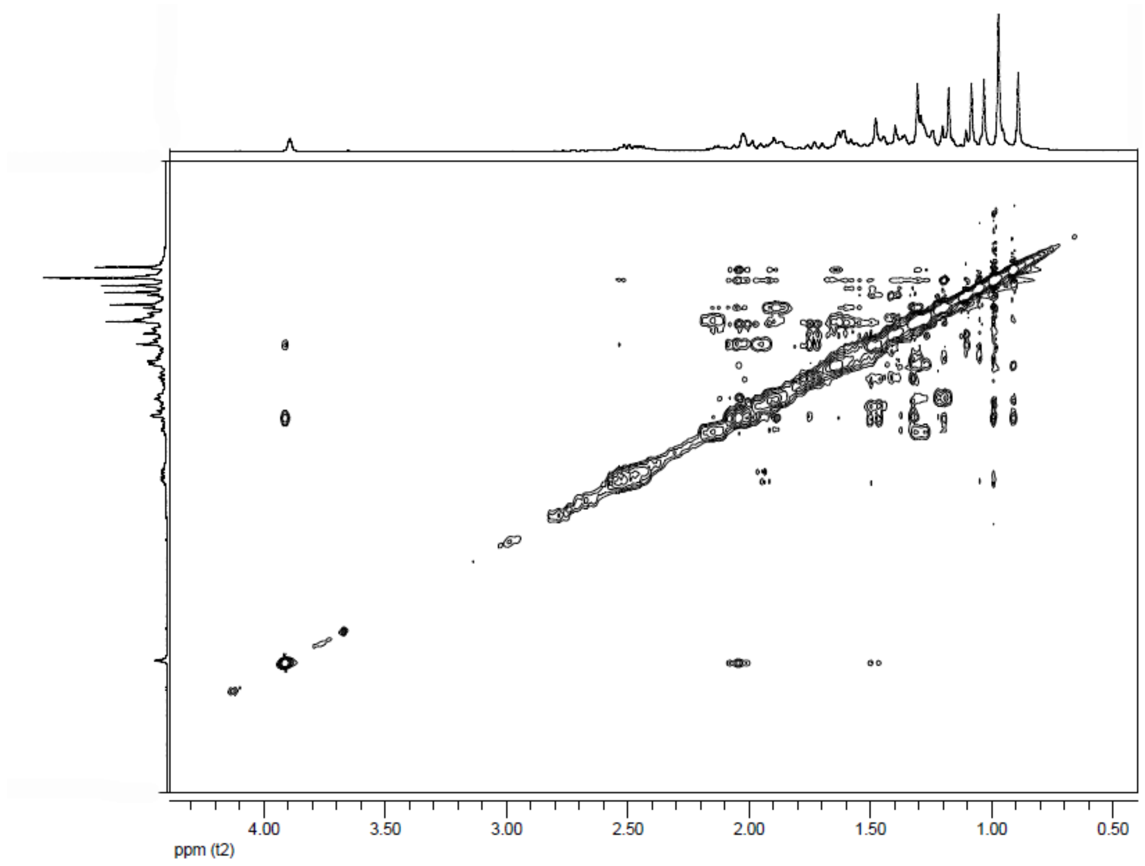

NOESY spectrum of compound **2** recorded in $CDCl_3$.

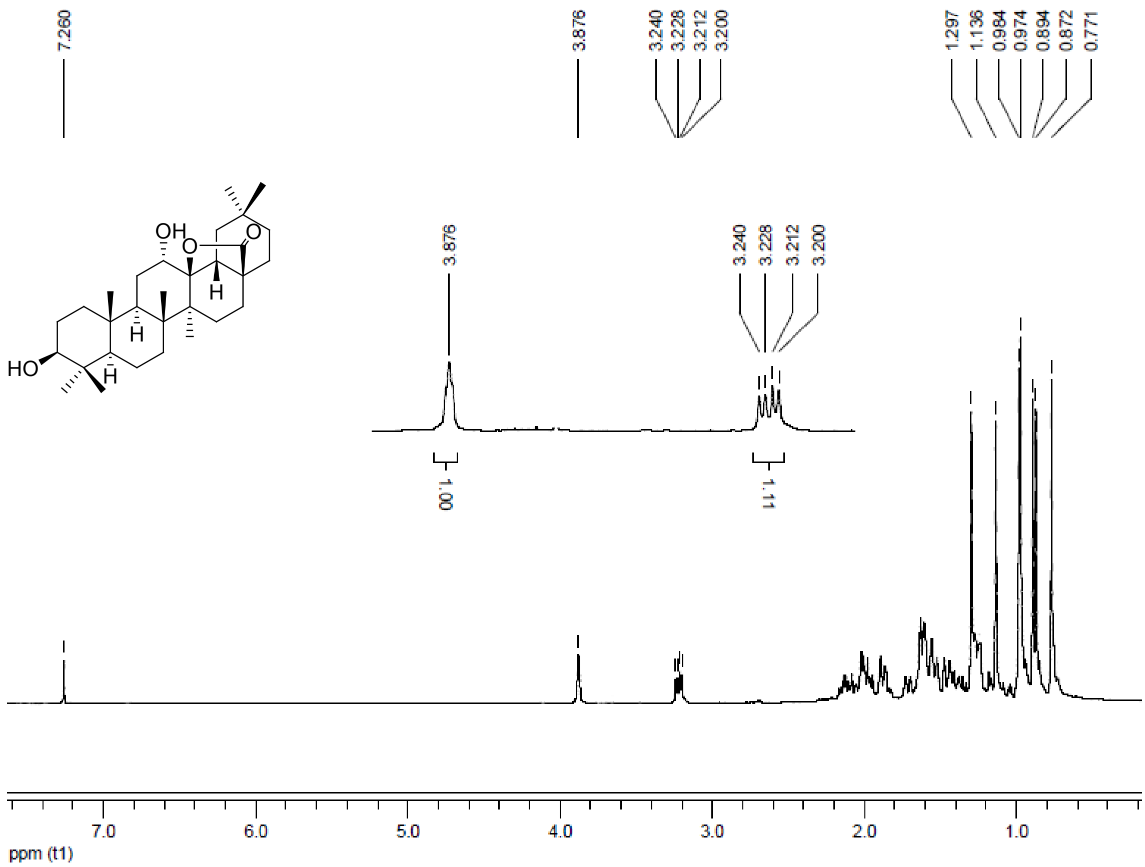

^{1}H NMR spectrum of compound **4** recorded in $CDCl_3$.

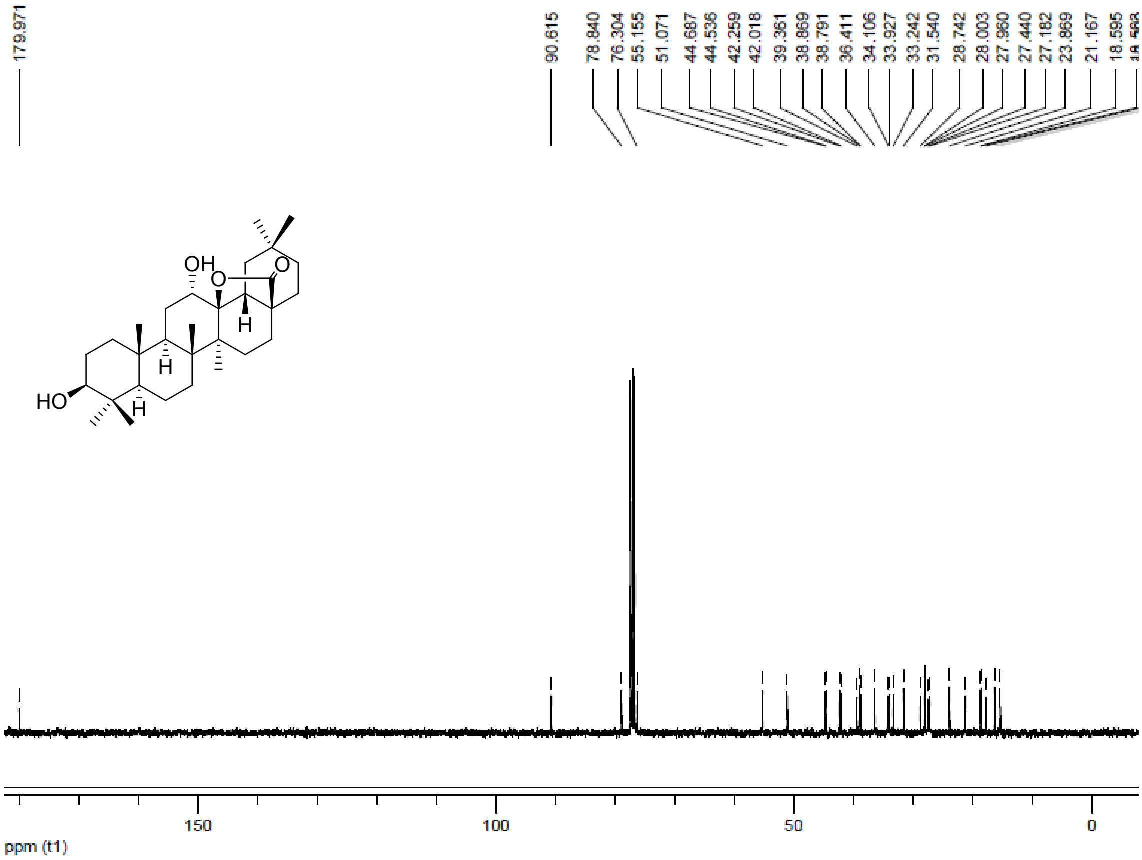

^{13}C NMR spectrum of compound **4** recorded in $CDCl_3$.

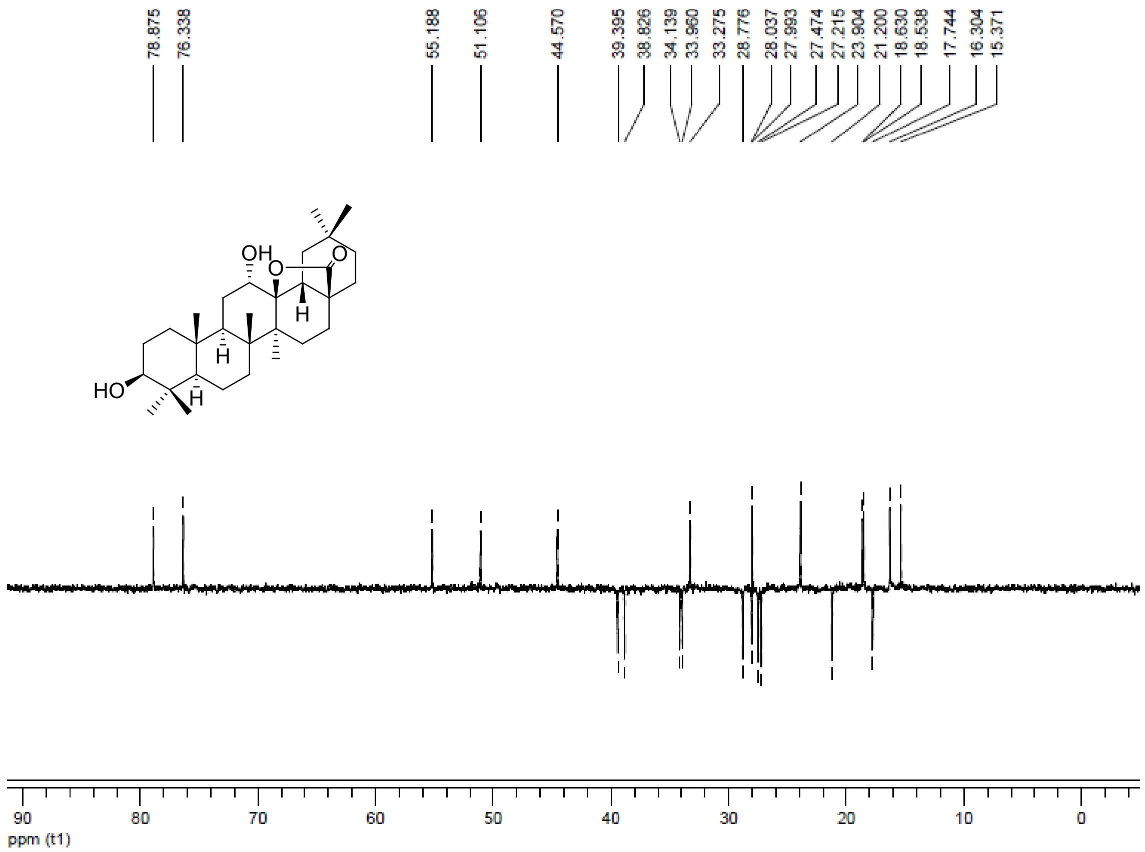

DEPT-135 spectrum of compound **4** recorded in $CDCl_3$.

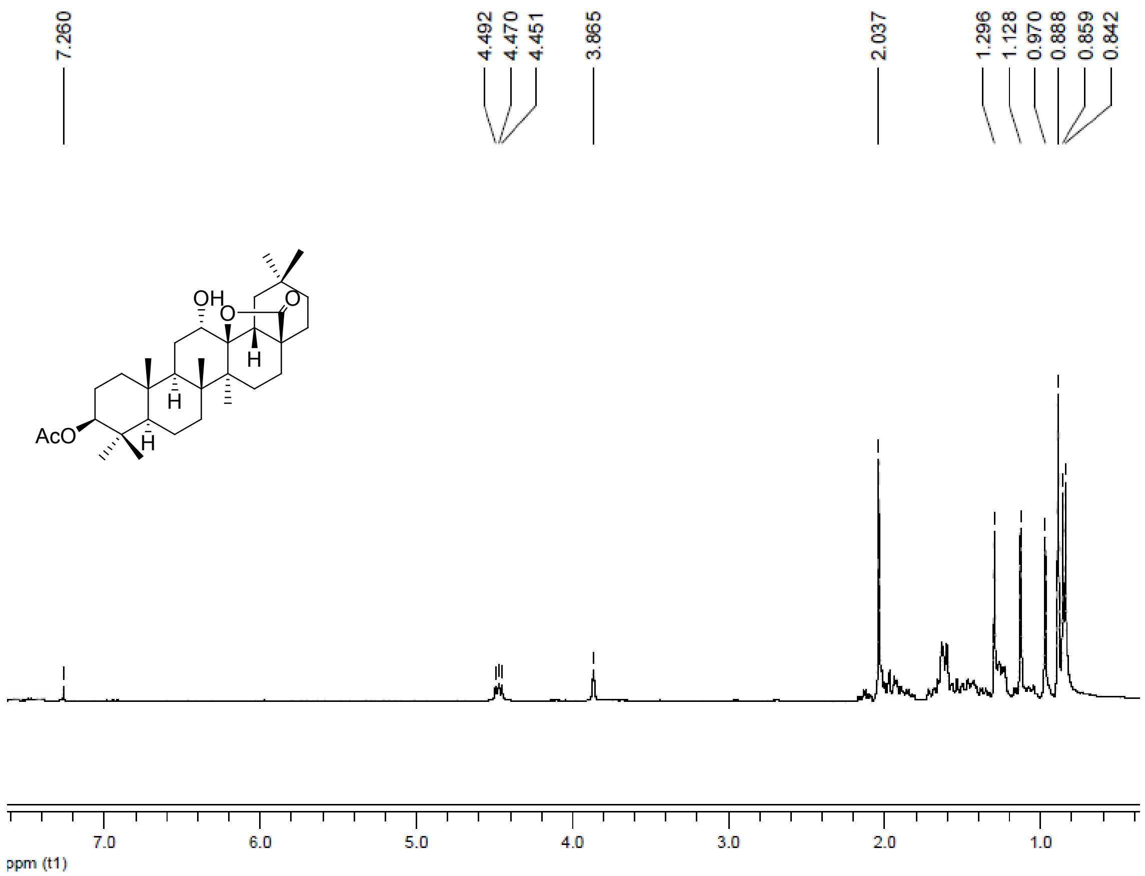

^{1}H NMR spectrum of compound **6** recorded in $CDCl_3$.

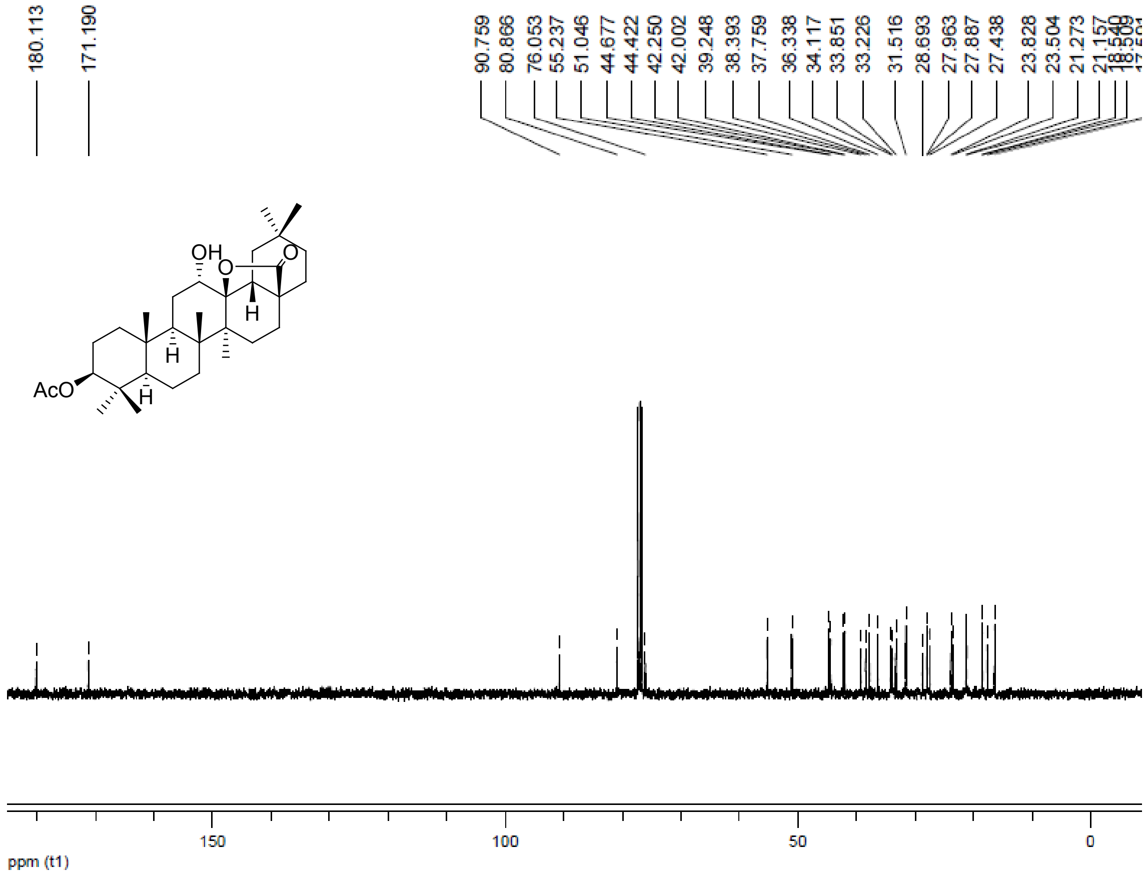

^{13}C NMR spectrum of compound **6** recorded in $CDCl_3$.

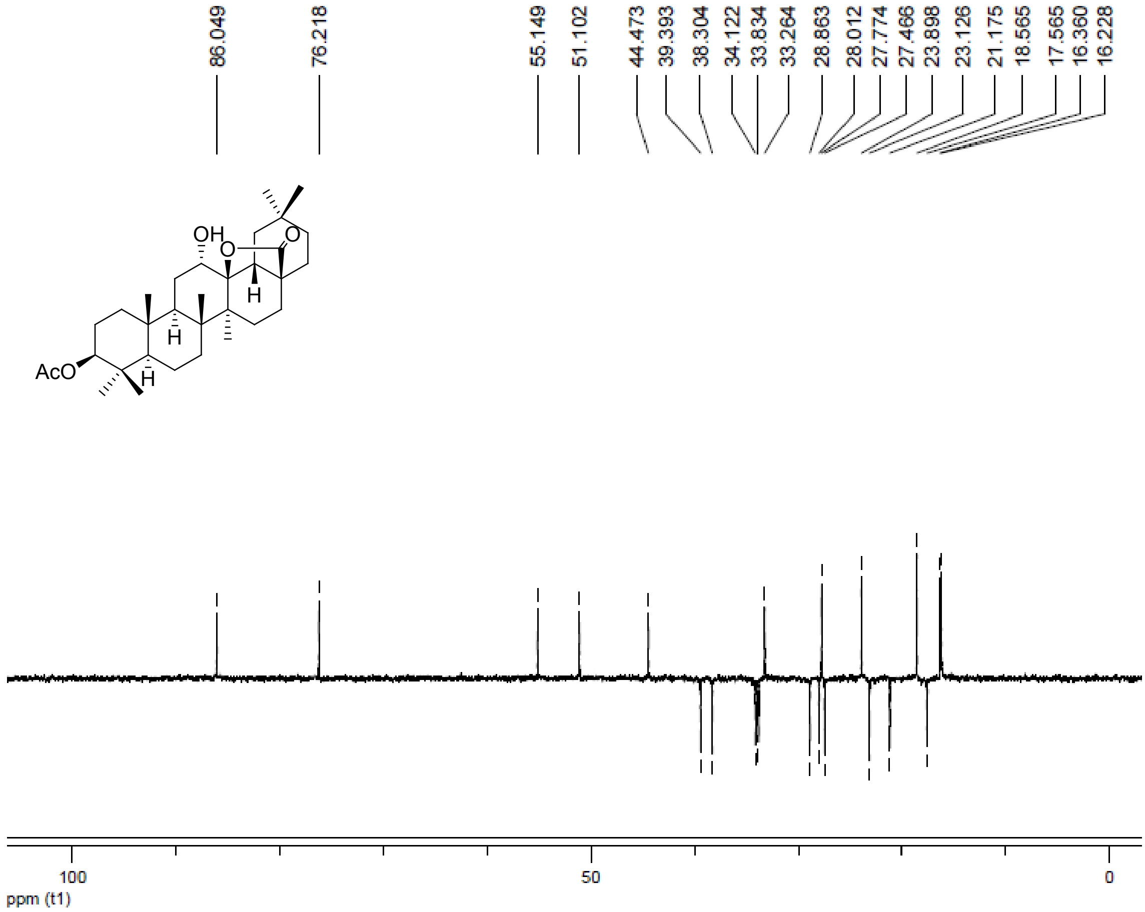

DEPT-135 spectrum of compound **6** recorded in $CDCl_3$.

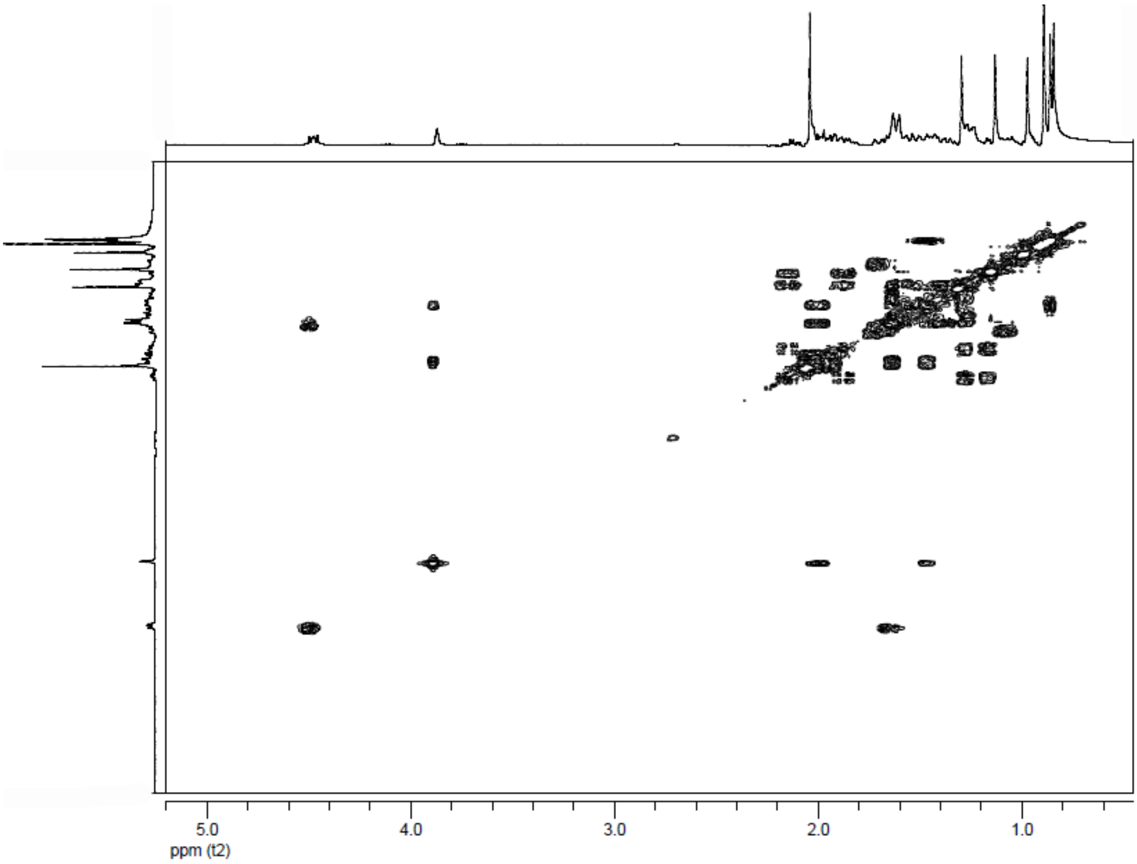

COSY spectrum of compound **6** recorded in $CDCl_3$.

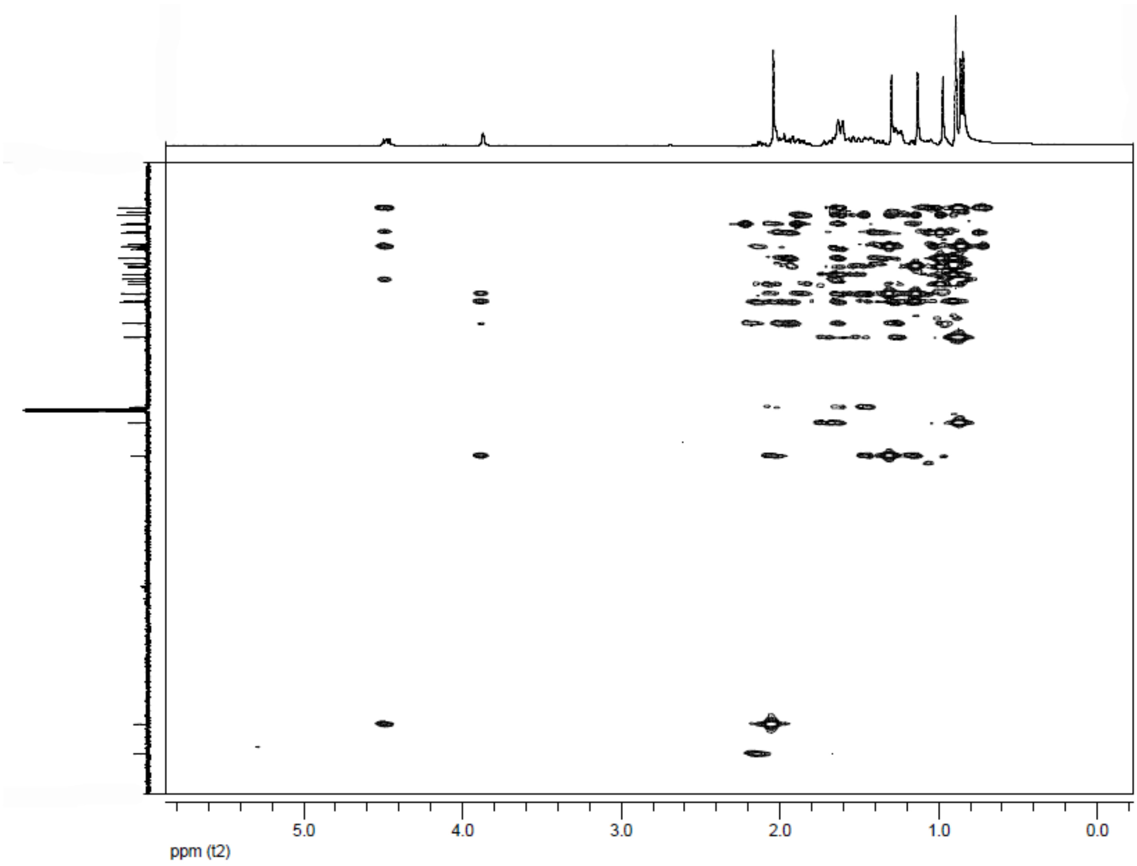

HMBC spectrum of compound **6** recorded in $CDCl_3$.

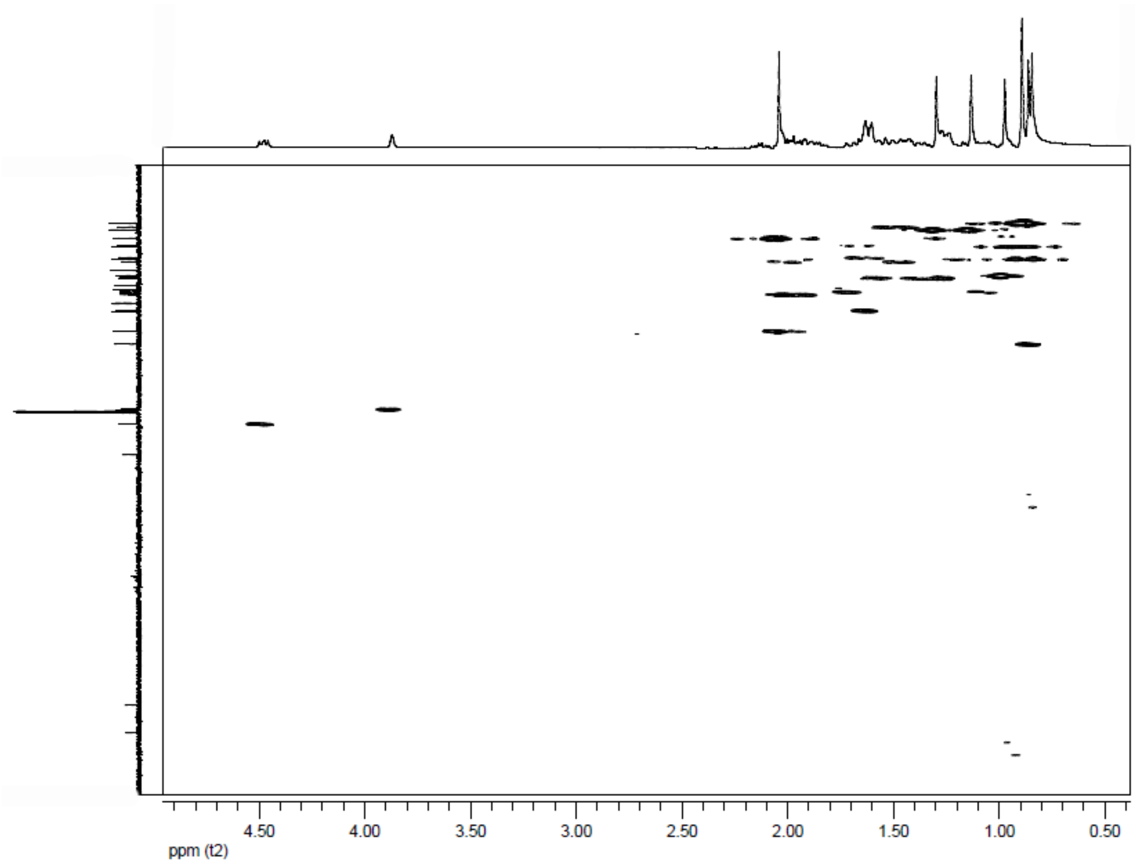

HMQC spectrum of compound **6** recorded in $CDCl_3$.

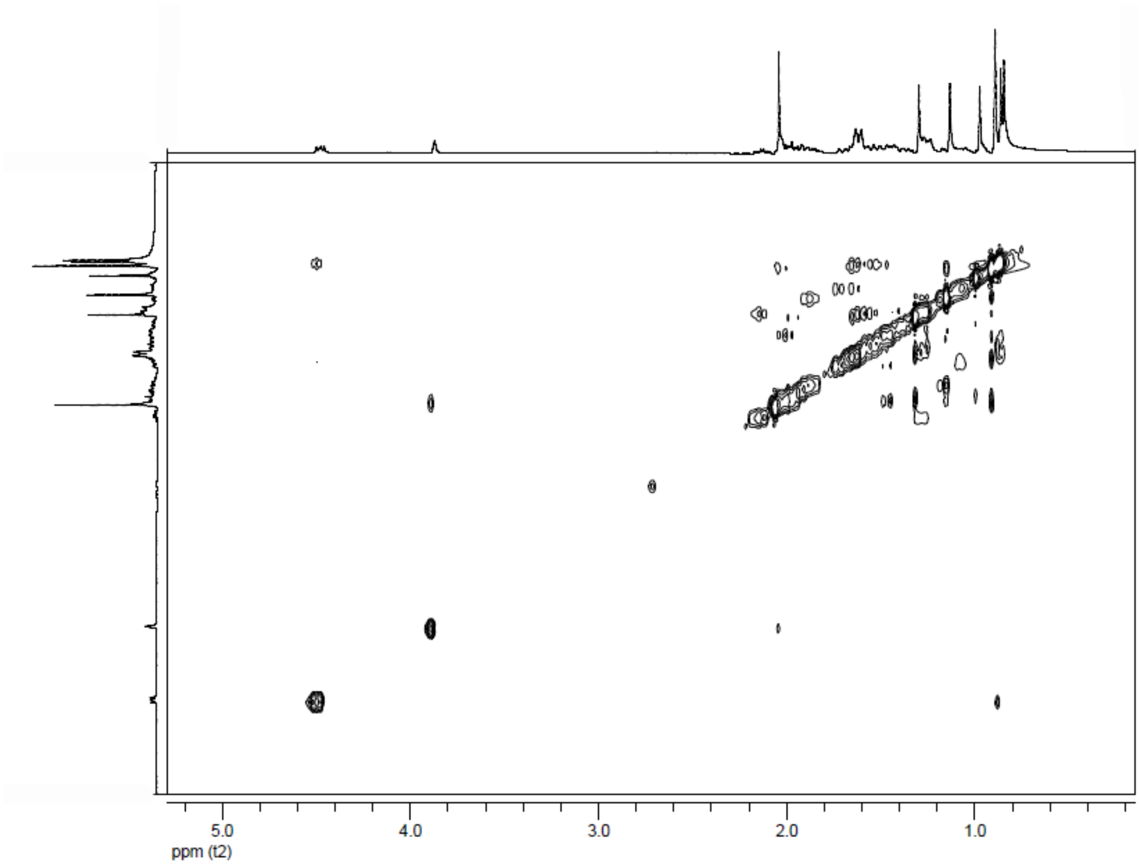

NOESY spectrum of compound **6** recorded in $CDCl_3$.

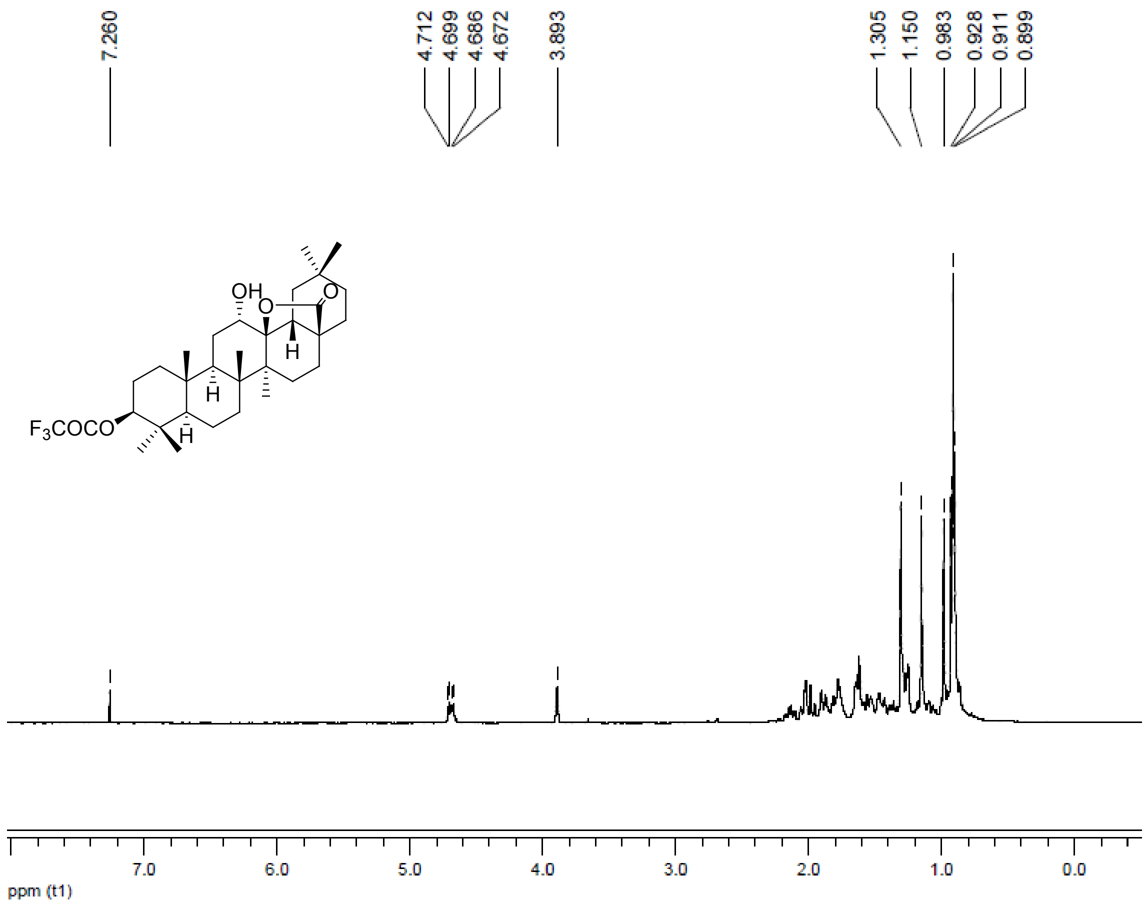

^{1}H NMR spectrum of compound **8** recorded in $CDCl_3$.

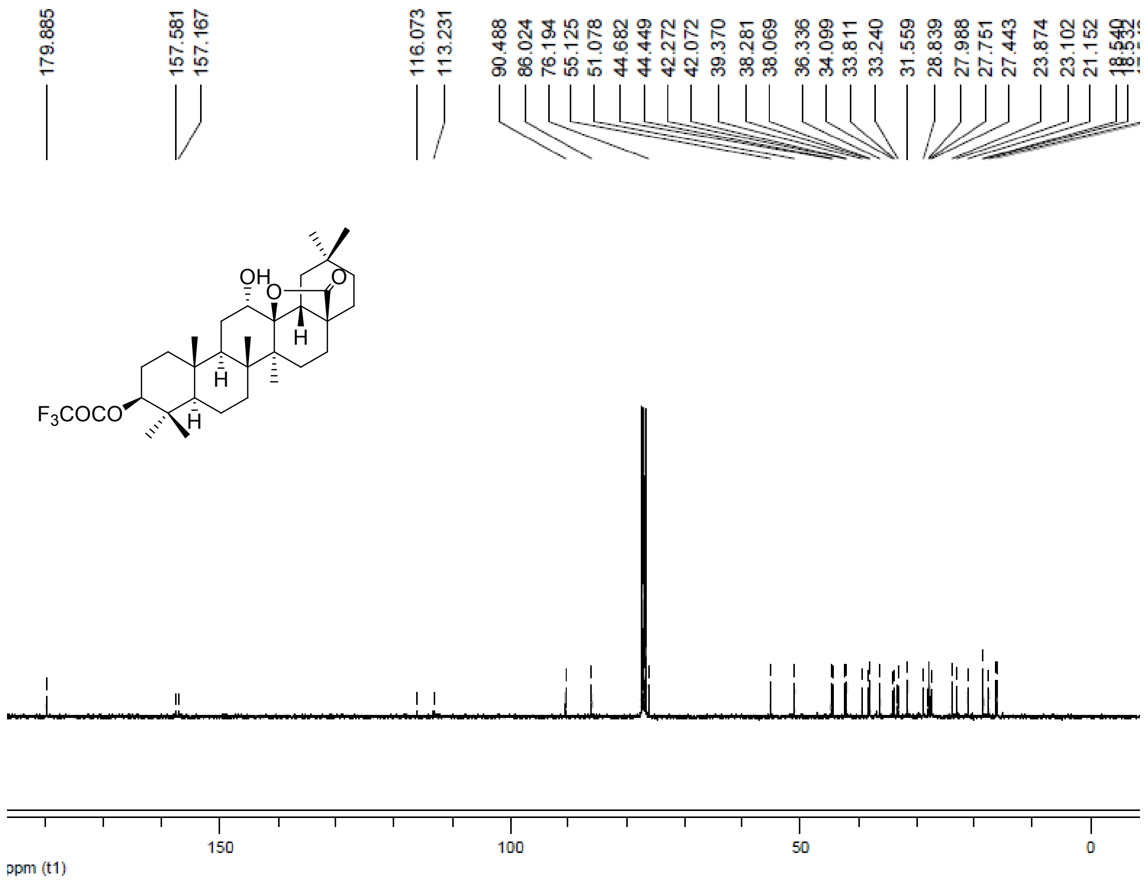

^{13}C NMR spectrum of compound **8** recorded in $CDCl_3$.

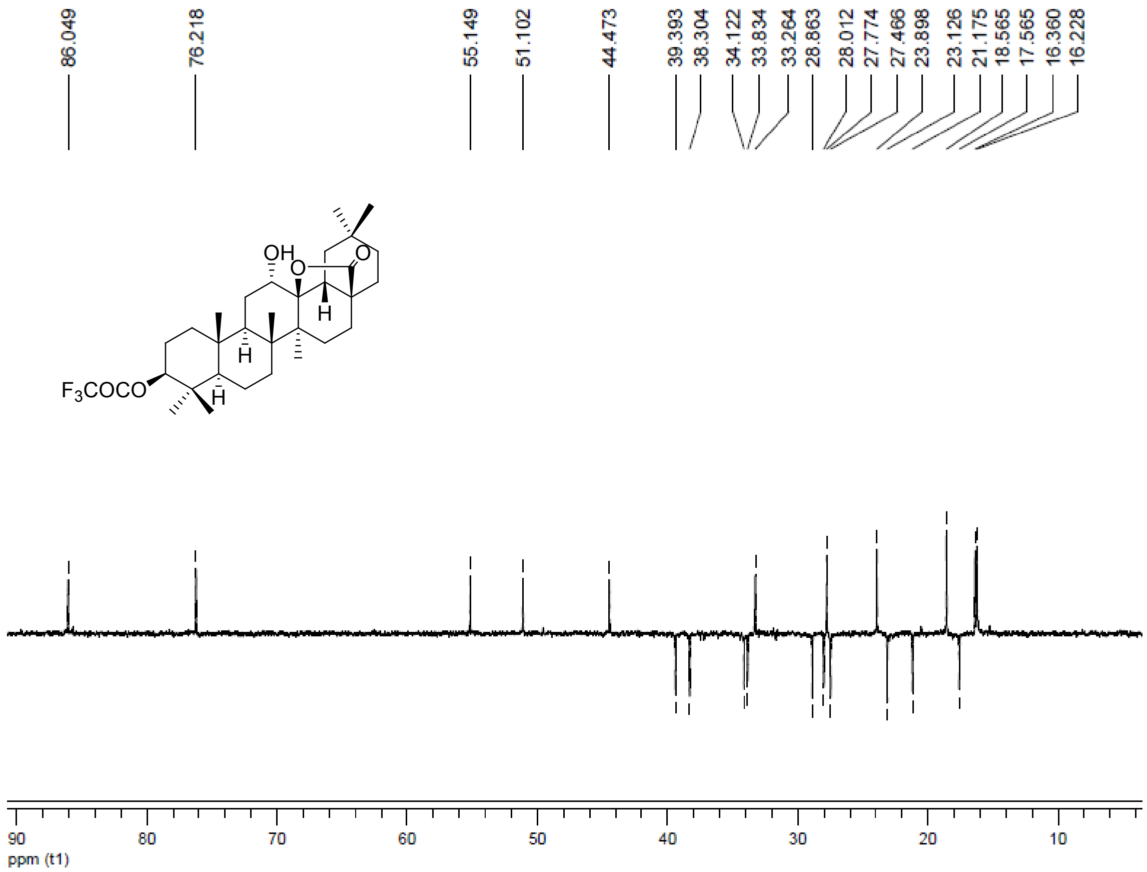

DEPT-135 spectrum of compound **8** recorded in $CDCl_3$.

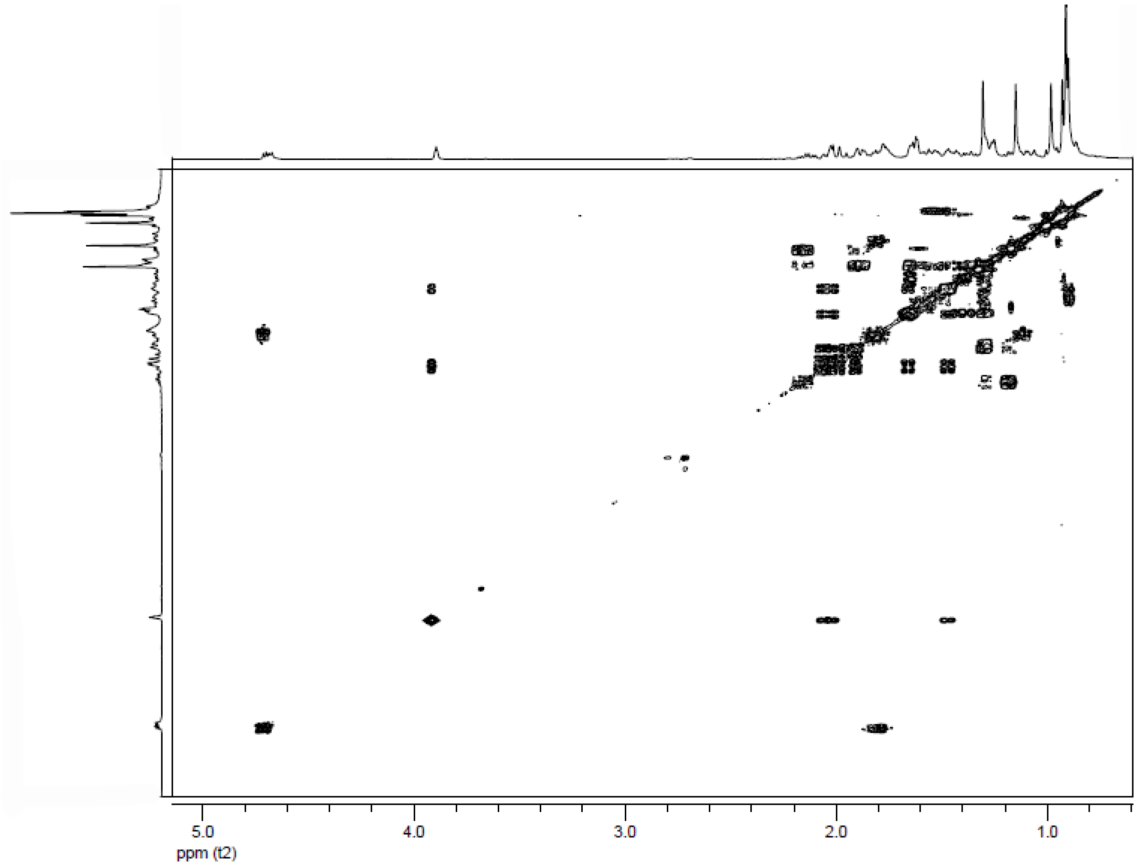

COSY spectrum of compound **8** recorded in $CDCl_3$.

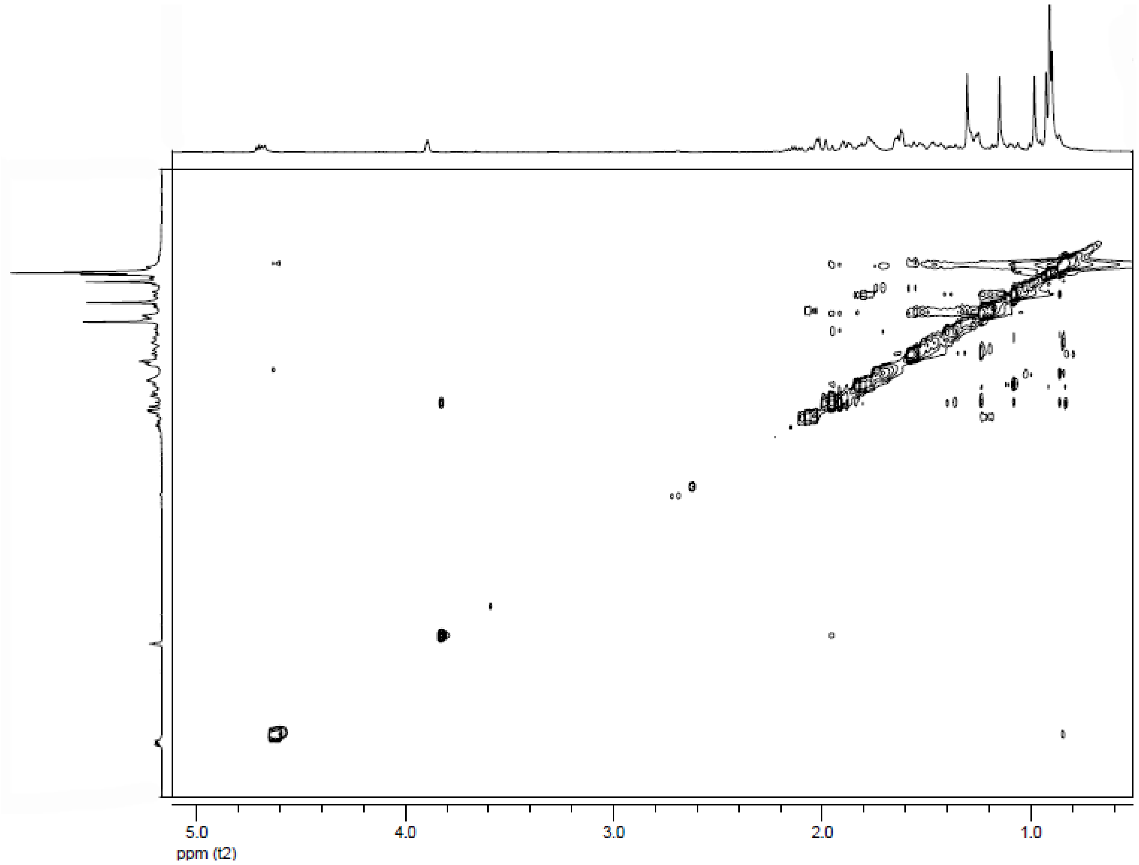

NOESY spectrum of compound **8** recorded in $CDCl_3$.

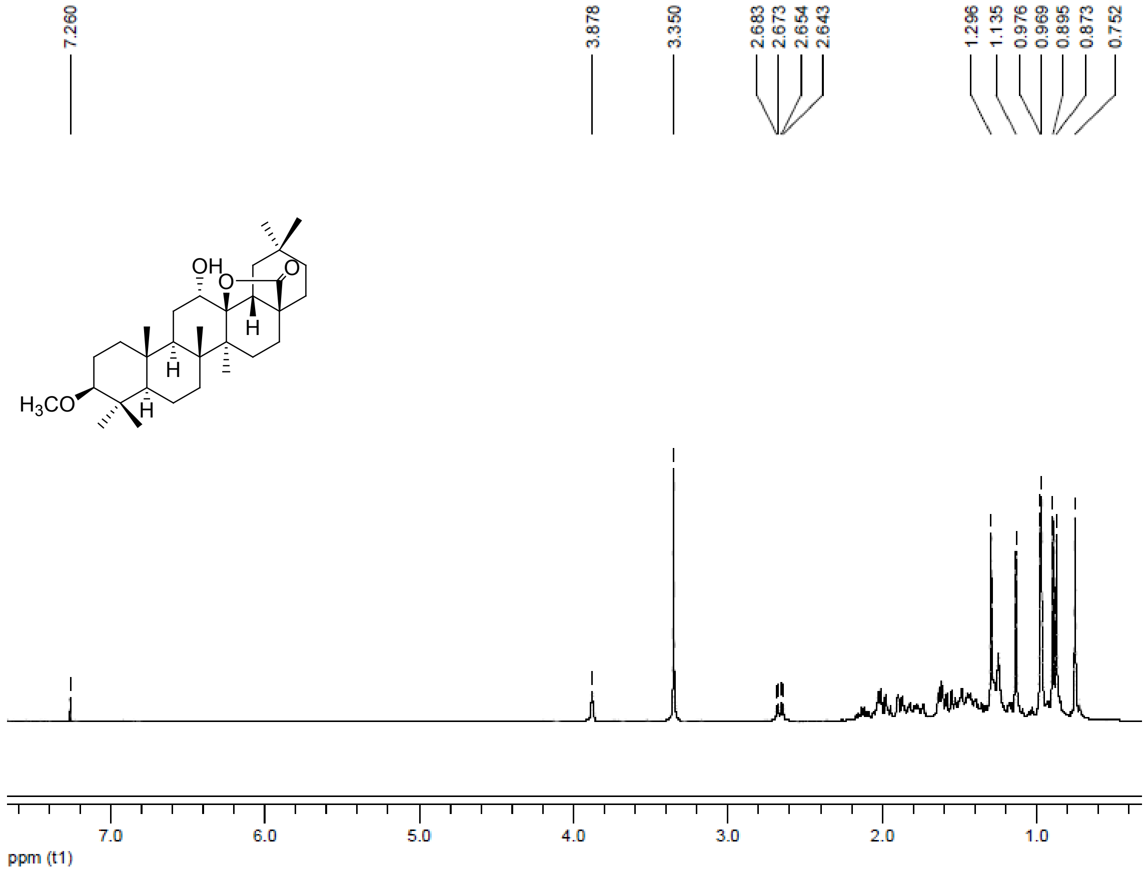

^{1}H NMR spectrum of compound **10** recorded in $CDCl_3$.

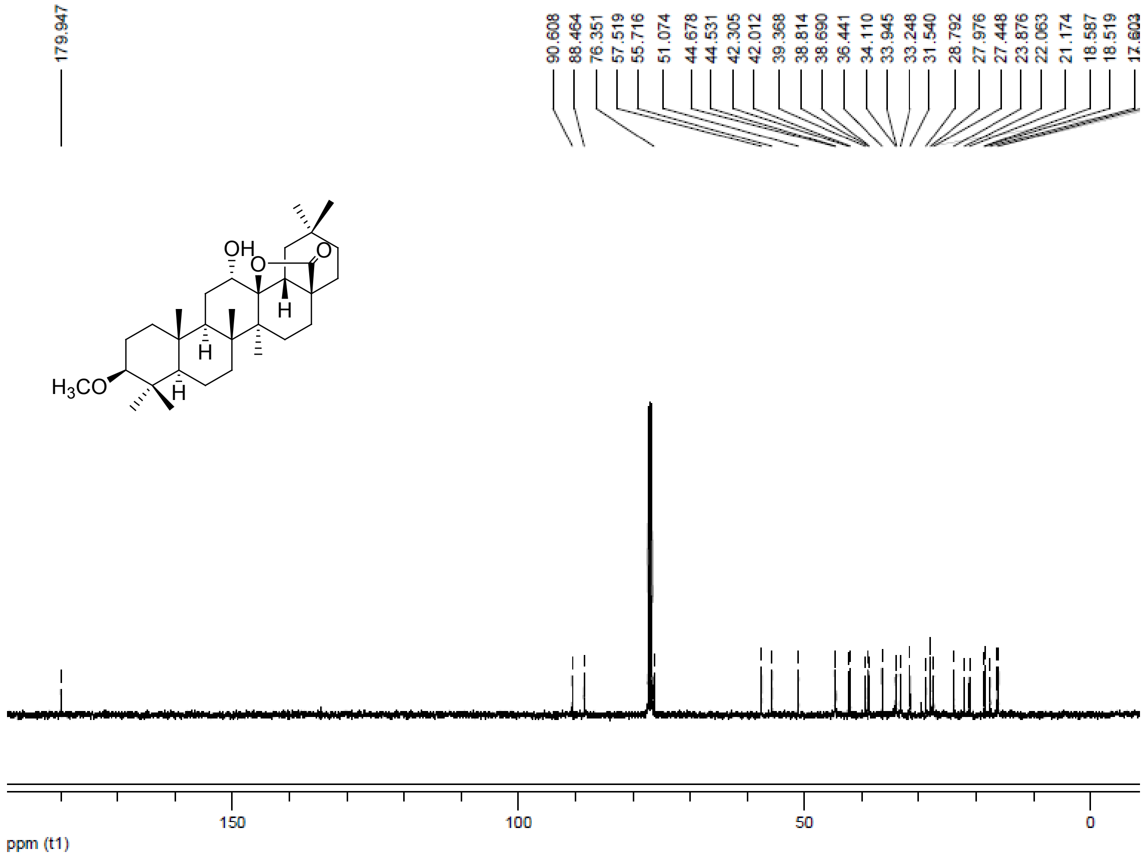

^{13}C NMR spectrum of compound **10** recorded in $CDCl_3$.

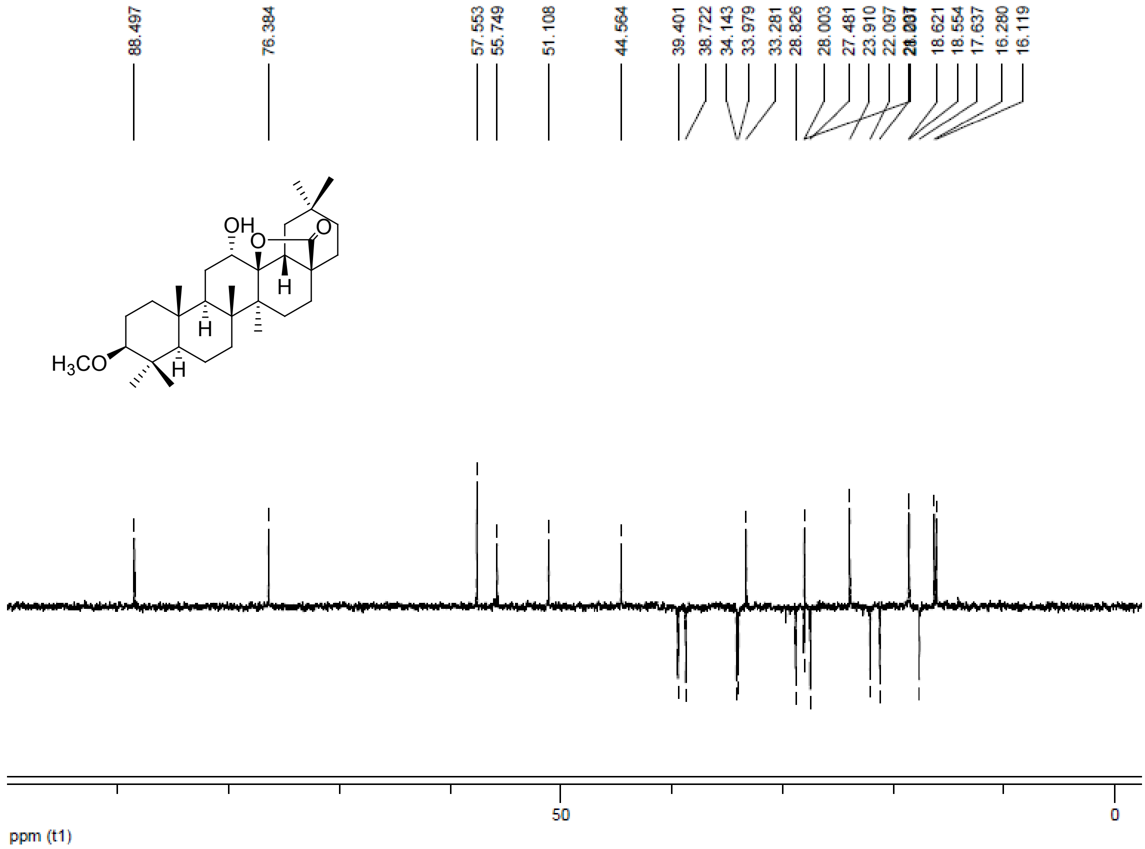

DEPT-135 spectrum of compound **10** recorded in $CDCl_3$.

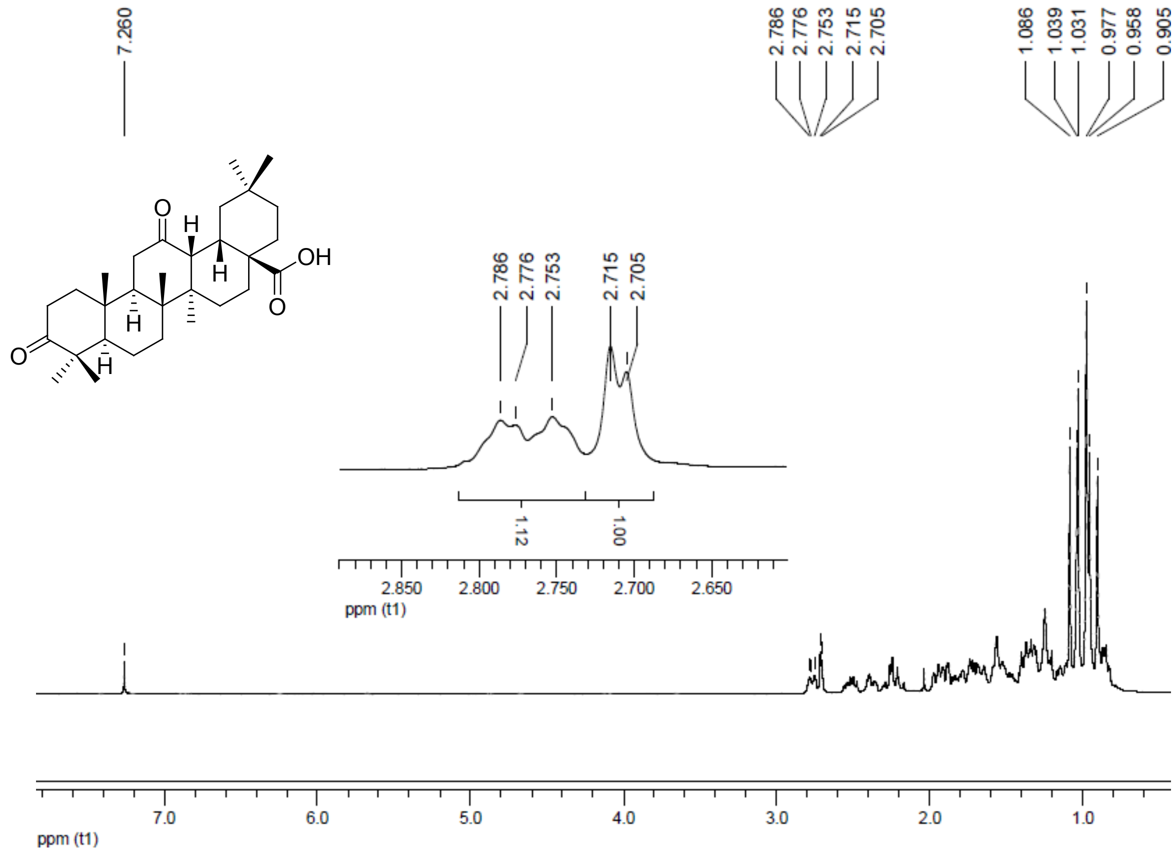

^{1}H NMR spectrum of compound **11** recorded in $CDCl_3$

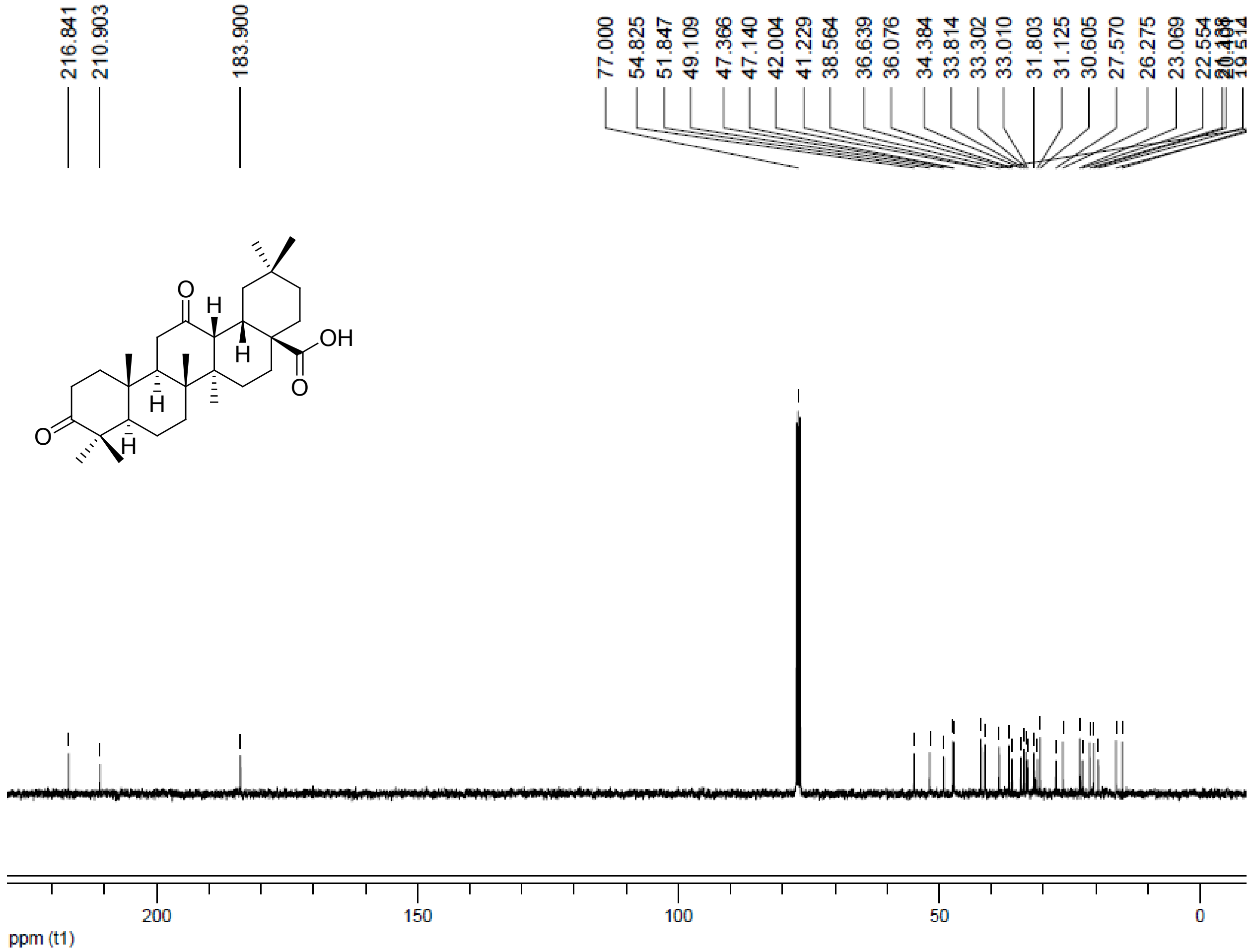

^{13}C NMR spectrum of compound **11** recorded in $CDCl_3$

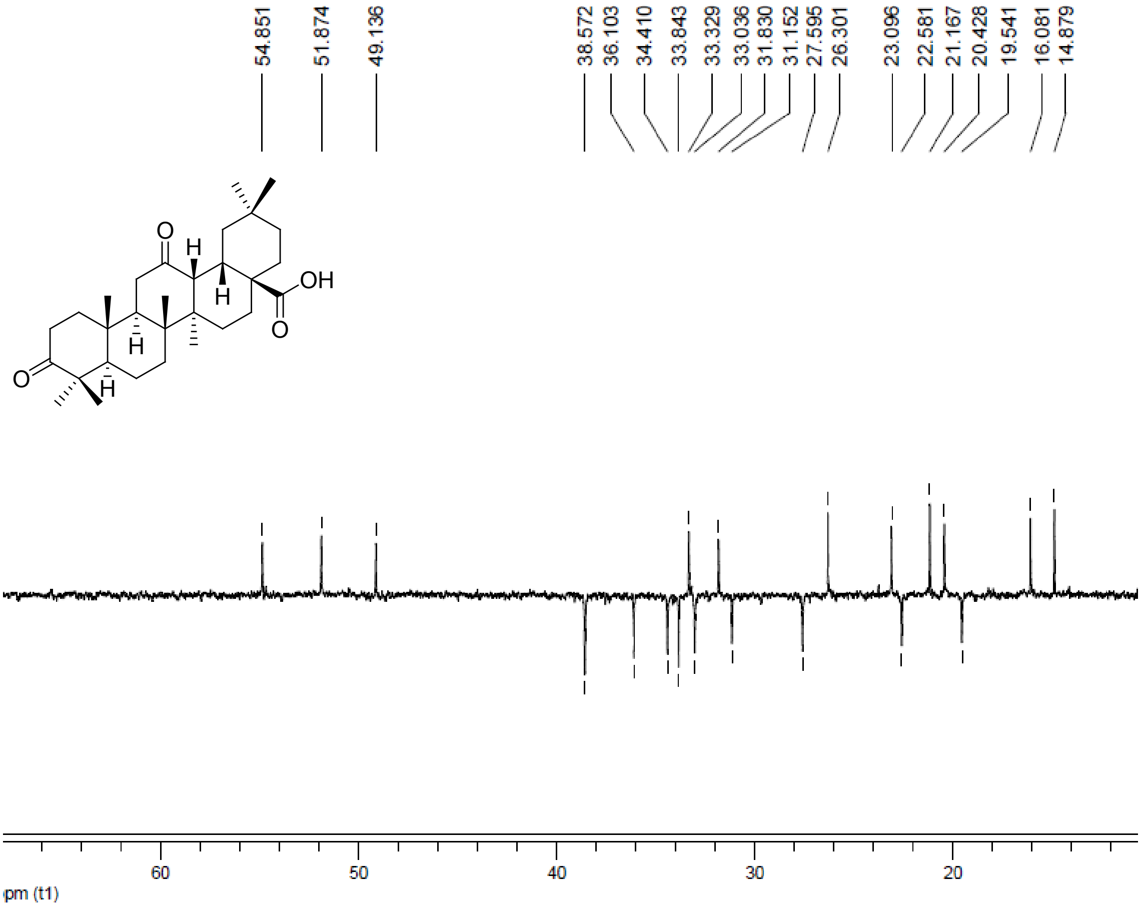

DEPT-135 spectrum of compound **11** recorded in $CDCl_3$

References

1. Konoike, T.; Takahashi, K.; Araki, Y.; Horibe, I. *J. Org. Chem.* **1997,** *62*, 960-966.
2. Zhu, Y.-M.; Shen, J.-K.; Wang, H.-K.; Cosentino, L. M.; Lee, K.-H. *Bioorg. Med. Chem.* **2001,** *11*, 3115-3118.
3. Salvador, J. A. R.; Moreira, V. M.; Pinto, R. M. A.; Leal, A. S.; Le Roux, C. *Adv. Synth. Catal.* **2011**, 353, 2637-2642.
4. Kwon, T. H.; Lee, B.; Chung, S. H.; Kim, D. H.; Lee, Y. S. *Bull. Korean Chem. Soc.* **2009,** *30*, 119-123.
5. Ali, M. S.; Jahangir, M.; ul Hussan, S. S.; Choudhary, M. I. *Phytochem.* **2002,** *60*, 295-299.
6. Hichri, F.; Ben Jannet, H.; Cheriaa, J.; Jegham, S.; Mighri, Z. *Compt. Rend. Chim.* **2003,** *6*, 473-483.
